# CYB5A promotes osteogenic differentiation of MC3T3-E1 cells through autophagy mediated by the AKT/mTOR/ULK1 signaling pathway

**DOI:** 10.1038/s41598-025-97086-0

**Published:** 2025-04-17

**Authors:** Yanjie Zhang, Jinmeng Li, Beibei Liu, Peilin Wang, Hanyu Xiao, Qingfu Wang, Ruixin Li, Jian Zhang

**Affiliations:** 1 Department of Oral Implantology, Tianjin Stomatological Hospital, School of Medicine, Nankai University, Tianjin, 300041 China; 2 Tianjin Key Laboratory of Oral and Maxillofacial Function Reconstruction, Tianjin, 300041 China; 3 Department of Oral Mucosal Diseases, Tianjin Stomatological Hospital, School of Medicine, Nankai University, Tianjin, 300041 China

**Keywords:** Osteogenic differentiation, CYB5A, Autophagy, Bone disorder, Cell biology, Medical research, Molecular medicine

## Abstract

Bone metabolism involves complex genetic and cellular processes. While many advances have been made in understanding the molecular mechanisms of osteogenic differentiation, many aspects remain to be fully elucidated. This study investigated the role of CYB5A in promoting osteogenic differentiation of MC3T3-E1 cells and explored the influence of autophagy via the AKT/mTOR/ULK1 signaling pathway. CYB5A expression during osteogenesis was analyzed through bioinformatics, quantitative reverse transcription polymerase chain reaction, and Western blotting. CYB5A was overexpressed or knocked down via plasmid or small interfering RNA transfection, and its effects on cell proliferation, migration, and differentiation were evaluated. Results showed that CYB5A expression increased during differentiation without affecting proliferation. However, CYB5A significantly enhanced cell differentiation by stimulating autophagy, as indicated by an increased ratio of the autophagic marker LC3-II/LC3-I and reduced levels of P62. Mechanistically, CYB5A modulates autophagy by activating ULK1 and reducing active mTOR phosphorylation. Autophagy inhibitors and activators confirmed that the AKT/mTOR/ULK1 pathway mediates CYB5A’s regulatory effects on osteogenesis. This study reveals that CYB5A positively regulates osteogenic differentiation through autophagy, offering insights into bone metabolism mechanisms. These findings suggest that CYB5A is a promising therapeutic target for managing bone metabolic disorders.

## Introduction

The human skeleton maintains a dynamic balance between osteoblast-mediated bone formation and osteoclast-mediated bone resorption^1^. Intrinsic or extrinsic factors, such as malnutrition, medications, aging, endocrine dysfunction, chronic inflammation, and genetics, may disrupt this balance, leading to bone loss, insufficiency, and bone metabolic diseases. These conditions include osteolysis, osteoporosis, osteoarthritis, rheumatoid arthritis, and an increased risk of fractures, which significantly impair patients’ quality of life^2^. Osteogenic differentiation is regulated by multiple interacting genes and cellular activities. The molecular mechanisms governing this process are highly complex, and many remain to be fully understood, requiring further investigation^3^.

Autophagy supplies energy and essential materials for eukaryotic cell proliferation, differentiation, and maturation by degrading intracellular proteins and damaged organelles^4^. Basal autophagy is essential for maintaining osteoblast, osteoclast, and osteocyte function. During mineralization, osteoblasts often form autophagy-like vesicles in the cytoplasm, and both osteogenic differentiation and mineralization are associated with autophagy upregulation^5^. Orthopedic materials or drugs have been used to enhance autophagy in osteoblasts, promoting skeletal health^6–8^. Impaired autophagy in osteoblasts reduces new bone production and disrupts the dynamic balance between osteoblasts and osteoclasts^9,10^. Similarly, autophagy inhibition in osteocytes leads to osteocyte senescence^11,12^.

The regulation of bone metabolism by autophagy involves key proteins and pathways, with numerous studies demonstrating the crucial role of the mTOR/ULK1 pathway in promoting osteogenic differentiation of osteoblasts^8^. Specifically, ULK1, a core autophagy-related (ATG) protein, is inhibited by mTOR. When mTOR inhibition is relieved, ULK1 initiates autophagy by recruiting other ATG proteins to autophagy formation sites^9^. In contrast, mTOR acts as a primary autophagy inhibitor. Both AKT and ERK1/2 activate mTOR, suppressing ULK1 and hindering cellular autophagy downstream of mTOR^13^. Furthermore, autophagy regulates bone marrow-derived mesenchymal stem cell (BMSC) osteogenic differentiation in a time-dependent manner via the AKT/mTOR pathway^14^. Therefore, modulating autophagy offers new strategies and potential therapeutic targets for bone metabolic diseases by influencing osteoblast function.

The protein encoded by CYB5A is a membrane-bound cytochrome involved in several important redox reactions, including cytochrome P450 and methemoglobin reduction^15^. Reduced CYB5A expression is associated with steroid-induced premenopausal osteoporosis in women^16^. Similarly, in an osteoporotic mouse model of liver dysfunction, CYB5A activity is diminished^17^, suggesting a role in osteogenic differentiation. Recent studies have indicated that CYB5A participates in the early phases of autophagy. It induces the formation of intracellular autophagic vesicles, lysosome accumulation, and upregulating intracellular ATG proteins, such as LC3-II, ATG5, ATG7, ATG9A, and ATG16L2, thereby increasing autophagy levels^18^. LC3-II and ATG5 expression during osteogenic differentiation is closely linked to bone formation and mineralization^19,20^, whereas ATG7 is essential for cell survival and function, with its deficiency reducing bone formation^21^. However, the mechanism by which CYB5A regulates osteogenic differentiation and the precise role of autophagy in this process remain unclear and require further investigation.

Therefore, we hypothesized that CYB5A regulates osteogenic differentiation by mediating autophagy. This study aimed to explore the role of CYB5A in the osteogenic differentiation of MC3T3-E1 cells and investigate the potential molecular mechanisms involved. The effect of CYB5A on MC3T3-E1 cell proliferation, migration, and osteogenic differentiation was also assessed. Subsequently, we analyzed the changes in ATG and AKT/mTOR/ULK1 pathway proteins. Finally, autophagy inhibitors and activators were used to confirm whether CYB5A promotes osteogenic differentiation through AKT/mTOR/ULK1-mediated autophagy.

## Material and methods

### Microarray analysis

The mRNA expression datasets during MC3T3-E1 osteogenic differentiation (GSE30393 and GSE46400) and hMSC osteogenic differentiation (GSE37558 and GSE80614) were obtained from the Gene Expression Omnibus (GEO) database (https://www.ncbi.nlm.nih.gov/geo/)^22,23^. Differences in CYB5A expression over time were analyzed and visualized using RStudio software (version 4.2.2, https://www.r-project.org/).

### Cell culture

MC3T3-E1 cells (National Infrastructure of Cell-line Resource, China) were cultured in a growth medium containing 10% fetal bovine serum (FBS, Gibco, United States), 1% penicillin/streptomycin (Gibco, United States), and MEM Alpha basal medium (α-MEM, Gibco, United States). The cell culture environment was maintained at 37 °C and 5% CO_2_ with fresh medium provided every 2 days. The osteogenic medium consists of 10 nM dexamethasone (Sigma-Aldrich, United States), 10 mM β-glycerophosphate (Sigma-Aldrich, United States), 0.2 mM ascorbic acid (Sigma-Aldrich, United States), and growth medium. To investigate autophagy in MC3T3-E1 cells, 3-methyladenine (3-MA, MedChemExpress, United States), rapamycin (RA, MedChemExpress, United States), and Chloroquine phosphate (CQ, MedChemExpress, United States) were added to the osteogenic induction solution at concentrations of 10 mM, 50 nM, and 10 μM respectively.

### Plasmid and small interfering RNA (siRNA) transfection

Plasmids and siRNAs targeting CYB5A were constructed by Shanghai GenePharma Co., Ltd. (Shanghai, China). CYB5A was overexpressed using the pcDNA3.1 vector containing full-length CYB5A (CYB5A group), whereas the empty vector pcDNA3.1 (Vector group) served as a control. Si-CYB5A was used to knock down CYB5A (si-CYB5A group) and non-targeting si-NC (si-NC group) as a control. The specific plasmid or siRNA was transfected into MC3T3-E1 cells using Lipofectamine 3000 (ThermoFisher, United States), following the manufacturer’s instructions, and the cells were used for subsequent experiments 48 h post-transfection. The transfection efficiency of CYB5A in MC3T3-E1 cells was examined using qRT-PCR and Western blotting. MC3T3-E1 cells transfected with the pcDNA3.1 vector containing full-length CYB5A were cultured in an osteogenic medium containing 3-MA (3-MA group) or CQ (CQ group). MC3T3-E1 cells transfected with si-CYB5A were cultured in an osteogenic medium containing RA (RA group).

### Cell proliferation assay

Cell proliferation assays were conducted using the Cell Counting Kit-8 (CCK-8, Solarbio, China) and EdU detection kit (Epizyme, China). MC3T3-E1 cells were seeded in 96-well plates at a density of 1,000 cells/well and transfected 24 h post-seeding. After 1, 3, 5, and 7 days of culture, 100 μL of growth medium containing 10 μL of CCK-8 staining reagent was added to each well, followed by a 2-h incubation at 37 °C in a 5% CO_2_ environment. The absorbance was measured at 450 nm using a microplate spectrometer (Tecan, Switzerland). For the EdU assay, MC3T3-E1 cells were seeded in 24-well plates at a density of 3,000 cells/well and transfected 24 h later. A growth medium containing 10 μM EdU was added 48 h post-transfection. After 6 h of incubation, the cells were fixed and stained, per the manufacturer’s instructions. The stained cells were observed and photographed using an inverted fluorescence microscope (Nikon, Japan). Cell counts were obtained using ImageJ software, and cell proliferation was calculated as the ratio of EdU-positive to DAPI-positive cells.

### Cell migration assay

MC3T3-E1 cell migration was assessed using Transwell and scratch assays. Transwell chambers (6.5 mm diameter, 8.0 μm pore size, Corning, United States) were used to evaluate MC3T3-E1 cell migration. Transfected MC3T3-E1 cells were resuspended in FBS-free α-MEM and inoculated into the upper chamber at a density of 3 × 10^4^ cells/well, with the lower chamber containing medium supplemented with 10% FBS. After 24 h, cells in the upper chamber were removed using cotton swabs, and cells in the lower chamber were fixed with 4% paraformaldehyde. The shape and edges of the migrated MC3T3-31 cells were observed by enlarging the image, and the number of them was manually counted using ImageJ software. During the process of cell counting, migrated cells should be avoided being counted repeatedly. Stained cells were observed and photographed using an inverted fluorescence microscope. For the scratch assay, MC3T3-E1 cells were seeded into six-well plates and transfected 24 h post-seeding. After 48 h, a vertical scratch was made at the bottom of each well using a 200 μL sterile pipette tip, and cell debris was washed away with sterile phosphate buffer saline (PBS, Solarbio, China). Following the scratch, MC3T3-E1 cells were cultured in α-MEM without FBS. The scratch areas were observed and photographed under a microscope at 0, 24, and 36 h. ImageJ software was used to calculate the number of migrated cells and migrated area-to-scratch area ratio (mobility ratio).

### qRT-PCR

Total RNA was extracted from the MC3T3-E1 cells using the SteadyPure Universal RNA Extraction Kit (AG, China). The RNA concentration was measured using a spectrophotometer (Thermo, United States). RNA was reverse-transcribed into cDNA using the RNA Reverse Transcription Reagent Premix (AG, China), followed by amplification using a system from Bio-Rad (United States). Gene expression at the mRNA level was quantified using a real-time quantitative PCR instrument (Roche, Switzerland) with the SYBR Green Premix Pro Taq HS qPCR Kit (AG, China). Relative gene expression at the mRNA level was assessed by calculating 2^-ΔΔCT^ values using β-actin as the housekeeping gene. Primer sequences used for PCR amplification are listed in Supplementary Table 1.

### Western blotting

Total protein was extracted from the MC3T3-E1 cells using radioimmunoprecipitation assay (RIPA) lysis buffer (Beyotime, China) supplemented with protease inhibitors (Solarbio, China) and phosphatase inhibitors (Beyotime, China). Protein concentrations were determined using the BCA protein content assay kit (Solarbio, China). Subsequently, the extracted proteins were combined with 5X loading buffer (Beyotime, China) and heated at 95 °C for 5 min. Proteins were separated on 12% sodium dodecyl sulfate–polyacrylamide gel electrophoresis (SDS-PAGE) gels and subsequently transferred onto polyvinylidene fluoride (PVDF) membranes. Membranes were blocked for 1 h, incubated overnight at 4 °C with primary antibodies, and incubated the next day with secondary antibodies for 2 h at room temperature. The PVDF membrane was then immersed in an enhanced chemiluminescence (ECL) reagent (Beyotime, China) and visualized using the ChemiDoc Touch Imaging System (Bio-Rad, United States) to detect immunocomplexes. The results were analyzed using ImageJ software, with β-actin as the reference protein. Details of the antibodies used for Western blotting are listed in Supplementary Table 2.

### Alizarin Red S (ARS) staining and Alkaline phosphatase (ALP) staining

MC3T3-E1 cells were seeded in 12-well plates and transfected after 24 h. Osteogenic induction was initiated 48 h post-transfection. After 21 days, the cells were fixed with 4% paraformaldehyde and stained with 2% ARS staining solution (Beyotime, China). After 14 days of osteogenic induction, the cells were fixed in 4% paraformaldehyde and stained using the BCIP/NBT ALP Color Development Kit (Beyotime, China). Staining results were observed and photographed under a microscope.

### Statistical analysis

Experiments were repeated independently three times. Data are presented as mean ± standard deviation. Statistical differences between groups were determined using either Student’s t-test or one-way ANOVA. Statistical significance was set at p < 0.05. Data visualization was performed using GraphPad Prism 8.0 software.

## Results

### Endogenous CYB5A expression increases during MC3T3-E1 osteogenic differentiation

In this study, we analyzed variations in CYB5A expression in MC3T3-E1 cells at various stages of osteogenic differentiation using the GSE30393 and GSE46400 datasets. In the GSE30393 dataset, CYB5A expression in MC3T3-E1 cells was significantly upregulated after 5, 10, and 28 days of osteogenic induction compared with 2 days. Similarly, in the GSE46400 dataset, CYB5A expression in MC3T3-E1 cells was significantly upregulated after 14 days of osteogenic induction compared with no induction (see Supplementary Fig. 1). Additionally, we analyzed the variations in hMSCs at various stages of osteogenic differentiation using the GSE37558 and GSE80614 datasets. In the GSE37558 dataset, CYB5A expression in hMSCs was significantly upregulated after 2, 8, 12, and 25 days of osteogenic induction compared with 0 days. Similarly, in the GSE80614 dataset, CYB5A expression in hMSCs was significantly upregulated after 12 h, 1, 2, 3, and 4 days of osteogenic induction compared with 0 h (see Supplementary Fig. 2).

**Fig. 1:**
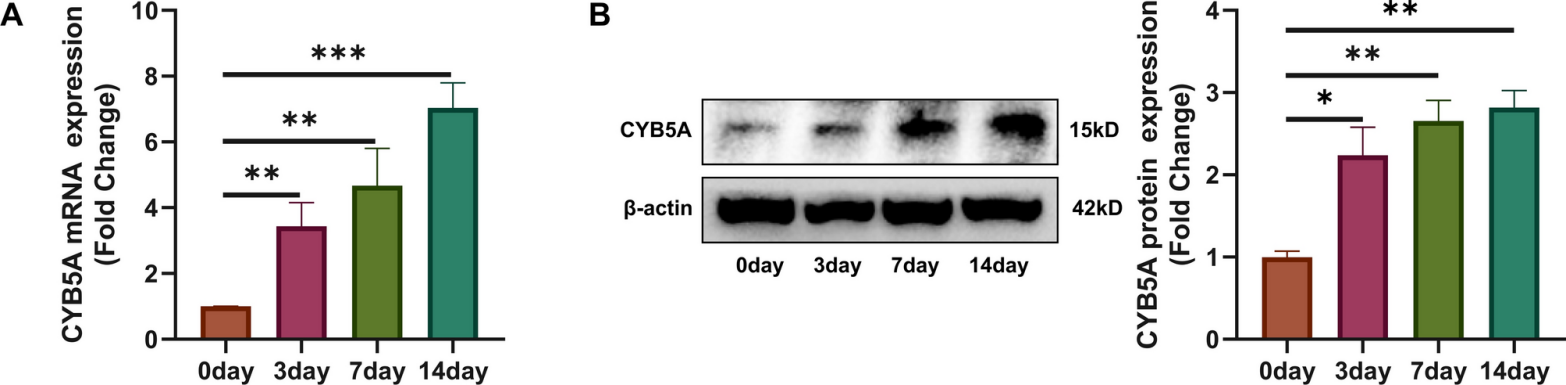
CYB5A expression during osteogenic differentiation of MC3T3-E1 cells. (**A**) qRT-PCR analysis showed that CYB5A mRNA expression was significantly upregulated during MC3T3-E1 cell osteogenic differentiation. (**B**) Western blot analysis and densitometric quantification confirmed that CYB5A protein levels increased during osteogenic differentiation. β-actin served as an internal control for normalization (n = 3). Statistical significance: *p < 0.05, **p < 0.01, ***p < 0.001.

To further elucidate the relationship between CYB5A and osteogenic differentiation in MC3T3-E1 cells, we assessed endogenous CYB5A expression at different stages of differentiation using qRT-PCR and Western blotting. CYB5A was significantly upregulated at both mRNA and protein levels after 3, 7, and 14 days of osteogenic differentiation compared with undifferentiated MC3T3-E1 cells (Fig. 1A, 1B). These results suggest that CYB5A plays a critical role in MC3T3-E1 osteogenic differentiation.

### Overexpression and knockdown efficiency of CYB5A in MC3T3-E1 cells

**Fig. 2:**
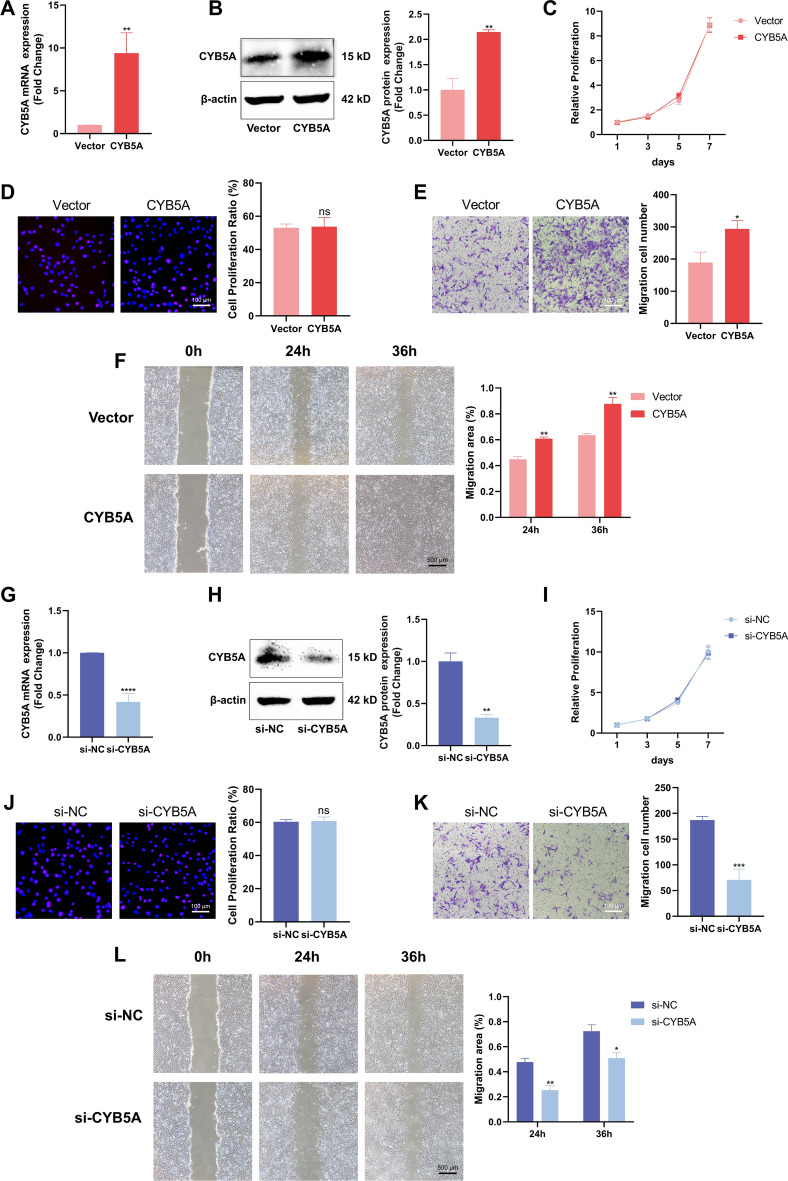
CYB5A did not affect the proliferation of MC3T3-E1 cells but promoted their migration. (**A**&**G**) qRT-PCR analysis confirmed the efficiency of CYB5A overexpression via plasmid and knockdown via siRNA in MC3T3-E1 cells. (**B**&**H**) Western Blotting analysis and quantification verified the effectiveness of CYB5A overexpression and knockdown at the protein level. (**C**&**I**) The cell viability of MC3T3-E1 cells was assessed using CCK‑8 assay. (**D**&**J**) Cell proliferation activity of MC3T3-E1 cells was evaluated using EdU assay, and the proportion of EdU-positive cells was calculated (ns = no significant difference, scale bar = 100 μm). (**E**&**K**) MC3T3-E1 cell migration was examined using the Transwell assay, and the number of migrated cells was quantified (scale bar = 100 μm). (**F**&**L**) The migration ability of MC3T3-E1 cells was analyzed using scratch assay, and the migration efficiency was calculated (scale bar = 500 μm). β-actin served as the internal control for normalization. Data are expressed as mean ± standard deviation (n = 3). Statistical significance: *p < 0.05, **p < 0.01, ***p < 0.001, ****p < 0.0001.

To investigate the effect of CYB5A on MC3T3-E1 cells, plasmid and siRNA were employed to overexpress or knock down CYB5A in MC3T3-E1 cells, respectively. The efficiency of overexpression and knockdown was evaluated by qRT-PCR and Western blotting. At 48 h post-transfection, CYB5A expression was significantly higher in the CYB5A group than in the Vector group (Fig. 2A, 2B). Similarly, at 48 h after siRNA transfection, CYB5A expression was significantly lower in the si-CYB5A group than in the si-NC group (Fig. 2G, 2H). These results demonstrate the successful overexpression and knockdown of CYB5A in MC3T3-E1 cells, with transfected cells used for subsequent experiments.

### CYB5A has no impact on MC3T3-E1 cell proliferation

The effects of CYB5A overexpression and knockdown on MC3T3-E1 cell proliferation were assessed using the CCK-8 and EdU cell proliferation assays. On days 1, 3, 5, and 7 after CYB5A overexpression or knockdown, no significant changes in MC3T3-E1 cellular activity were observed (Fig. 2C, 2I). Similarly, the EdU assay showed no significant changes in cell proliferation following CYB5A overexpression or knockdown (Fig. 2D, 2J). These findings indicate that CYB5A does not affect MC3T3-E1 cell proliferation.

### CYB5A positively regulates MC3T3-E1 cell migration

After culturing MC3T3-E1 cells in Transwell chambers for 24 h, migrated cells in the CYB5A group were significantly higher than those in the Vector group, whereas migrated cells in the si-CYB5A group were significantly lower than those in the si-NC group (Fig. 2E, 2K). These results suggest that CYB5A enhanced MC3T3-E1 cell migration. Furthermore, after 24 and 36 h of scratching, MC3T3-E1 cells in the CYB5A group exhibited a larger migration area compared with the Vector group, whereas those in the si-CYB5A group displayed a smaller migration area than those in the si-NC group (Fig. 2F, 2L).

### CYB5A stimulates osteogenic differentiation of MC3TE-31 cells

**Fig. 3:**
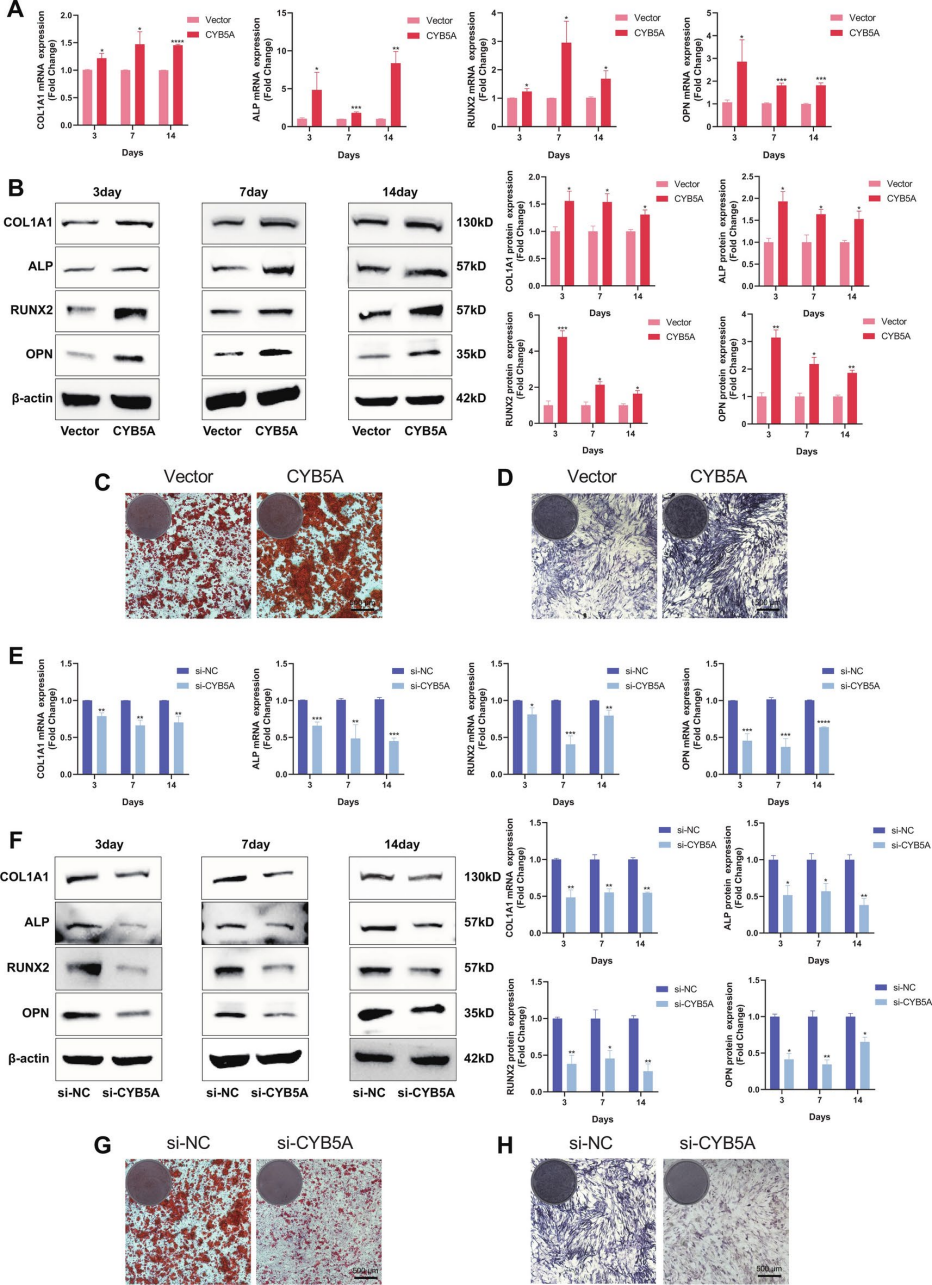
CYB5A promoted MC3T3-E1 cell osteogenic differentiation. (**A**, **B**, **E**, **F**) The expression of osteogenic markers (COL1A1, ALP, RUNX2, and OPN) was analyzed using qRT-PCR, Western Blotting, and quantification. (**C**&**G**) ARS staining was performed on MC3T3-E1 cells after 21 days of osteogenic induction. (**D**&**H**) ALP staining was conducted on MC3T3-E1 cells after 14 days of osteogenic induction. β-actin levels served as the internal normalization control. Data are presented as mean ± standard deviation (n = 3). Scale bar: 500 μm. Statistical significance: *p < 0.05, **p < 0.01, ***p < 0.001, ****p < 0.0001.

To assess the effect of CYB5A on MC3T3-E1 osteogenic differentiation, the levels of Collagen Type I Alpha 1 Chain (COL1A1), ALP (Biomineralization Associated), RUNX Family Transcription Factor 2 (RUNX2), and Osteopontin (OPN) were measured using qRT-PCR and Western blotting after 3, 7, and 14 days of osteogenic induction. The results showed that at all time points, the expression of COL1A1, ALP, RUNX2, and OPN in the CYB5A group was higher than in the Vector group (Fig. 3A, 3B), whereas their expression in the si-CYB5A group was lower than in the si-NC group (Fig. 3E, 3F).

To further investigate the role of CYB5A in osteogenic differentiation, osteogenic differentiation of MC3T3-E1 cells was evaluated using ALP and ARS staining on days 14 and 21. ARS staining revealed higher calcium nodules in the CYB5A group than in the Vector group (Fig. 3C), whereas calcium nodules in the si-CYB5A group were lower than in the si-NC group (Fig. 3G). In addition, ALP staining was significantly deeper in the CYB5A group compared with the Vector group (Fig. 3D), whereas staining in the si-CYB5A group was significantly lighter than in the si-NC group (Fig. 3H). These results suggest that CYB5A positively regulates the osteogenic differentiation of MC3T3-E1 cells.

### CYB5A stimulates autophagy in MC3T3-E1 cells

**Fig. 4:**
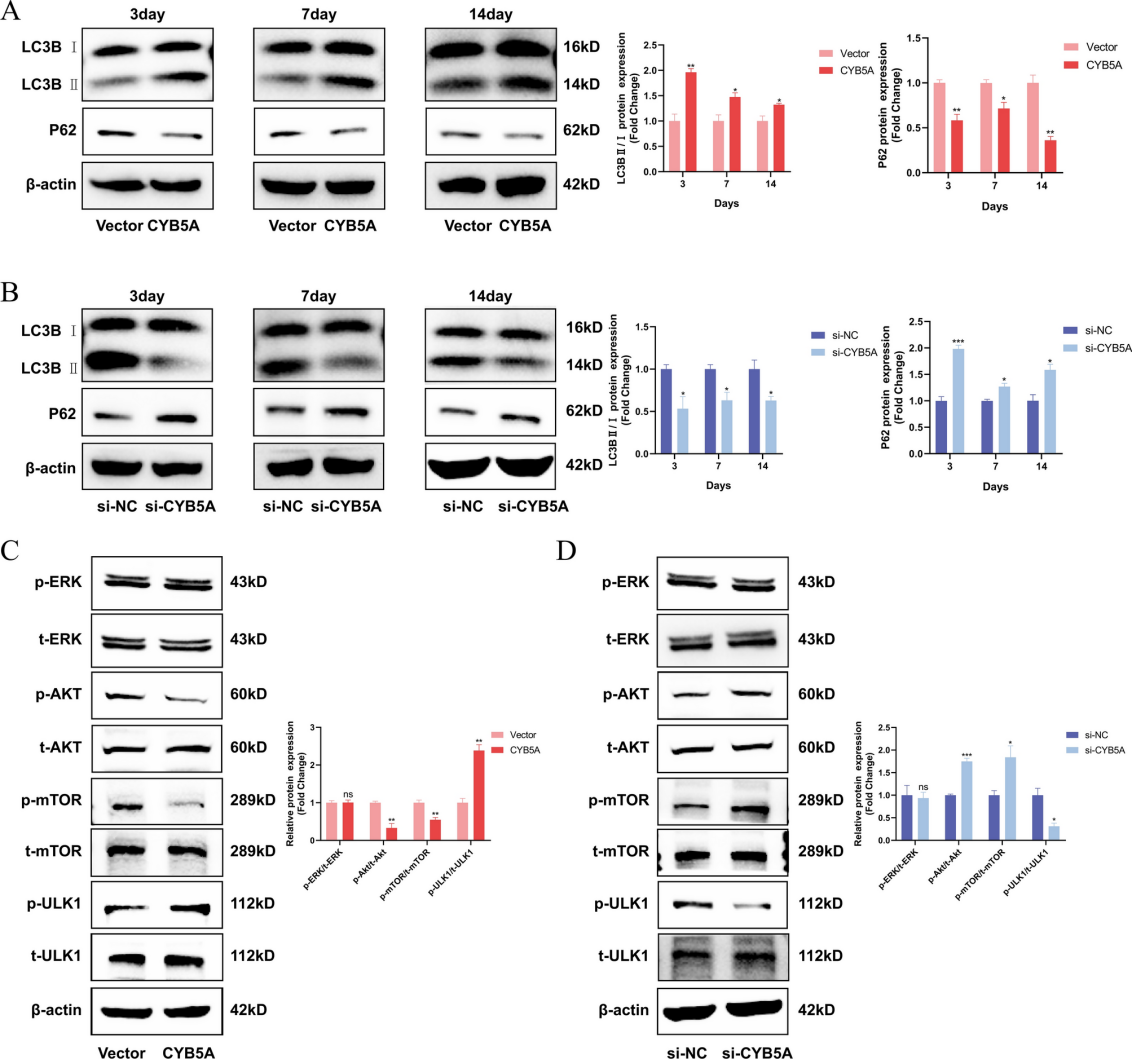
CYB5A activated autophagy via the AKT/mTOR/ULK1 signaling pathway in MC3T3-E1 cells. (**A**&**B**) Western Blotting and quantification were performed to assess LC3-I/II and P62 levels in MC3T3-E1 cells. (**C**&**D**) Western Blotting and quantification were used to analyze the levels of phosphorylated/total AKT, phosphorylated/total mTOR, and phosphorylated/total ULK1 in MC3T3-E1 cells. (β-actin levels were set as the internal normalized control, ns = no significant difference, n = 3, *p < 0.05, **p < 0.01, ***p < 0.001.)

To investigate whether CYB5A is associated with autophagy in MC3T3-E1 cells, changes in the autophagy proteins LC3-I/II and P62 were assessed by Western blotting after 3, 7, and 14 days of osteogenic differentiation. The results revealed that the LC3-II/LC3-I ratio of MC3T3-E1 cells was significantly higher, and P62 expression was lower in the CYB5A group compared with the Vector group (Fig. 4A). In contrast, the si-CYB5A group showed a lower LC3-II/LC3-I ratio and higher P62 expression than the si-NC group (Fig. 4B). These results suggest that autophagy is significantly altered during CYB5A-mediated regulation of MC3T3-E1 osteogenic differentiation.

The potential mechanism by which CYB5A regulates autophagy was investigated by analyzing protein alterations in the AKT/mTOR/ULK1 and ERK1/2 pathways using Western blotting after 3 days of osteogenic induction in MC3T3-E1 cells. The results demonstrated that neither CYB5A overexpression nor knockdown caused significant changes in the levels of ERK1/2 and p-ERK1/2 proteins in the ERK1/2 pathway. However, MC3T3-E1 cells in the CYB5A group exhibited lower levels of AKT and mTOR phosphorylation and higher levels of ULK1 protein phosphorylation than those in the Vector group (Fig. 4C). Conversely, MC3T3-E1 cells in the si-CYB5A group showed higher levels of AKT and mTOR phosphorylation and lower levels of ULK1 phosphorylation compared with the si-NC group (Fig. 4D). These findings suggest that CYB5A-mediated autophagy in MCET3-E1 cells is dependent on the AKT/mTOR/ULK1 pathway.

### CYB5A enhances osteogenic differentiation of MC3T3-E1 cells via autophagy

**Fig. 5:**
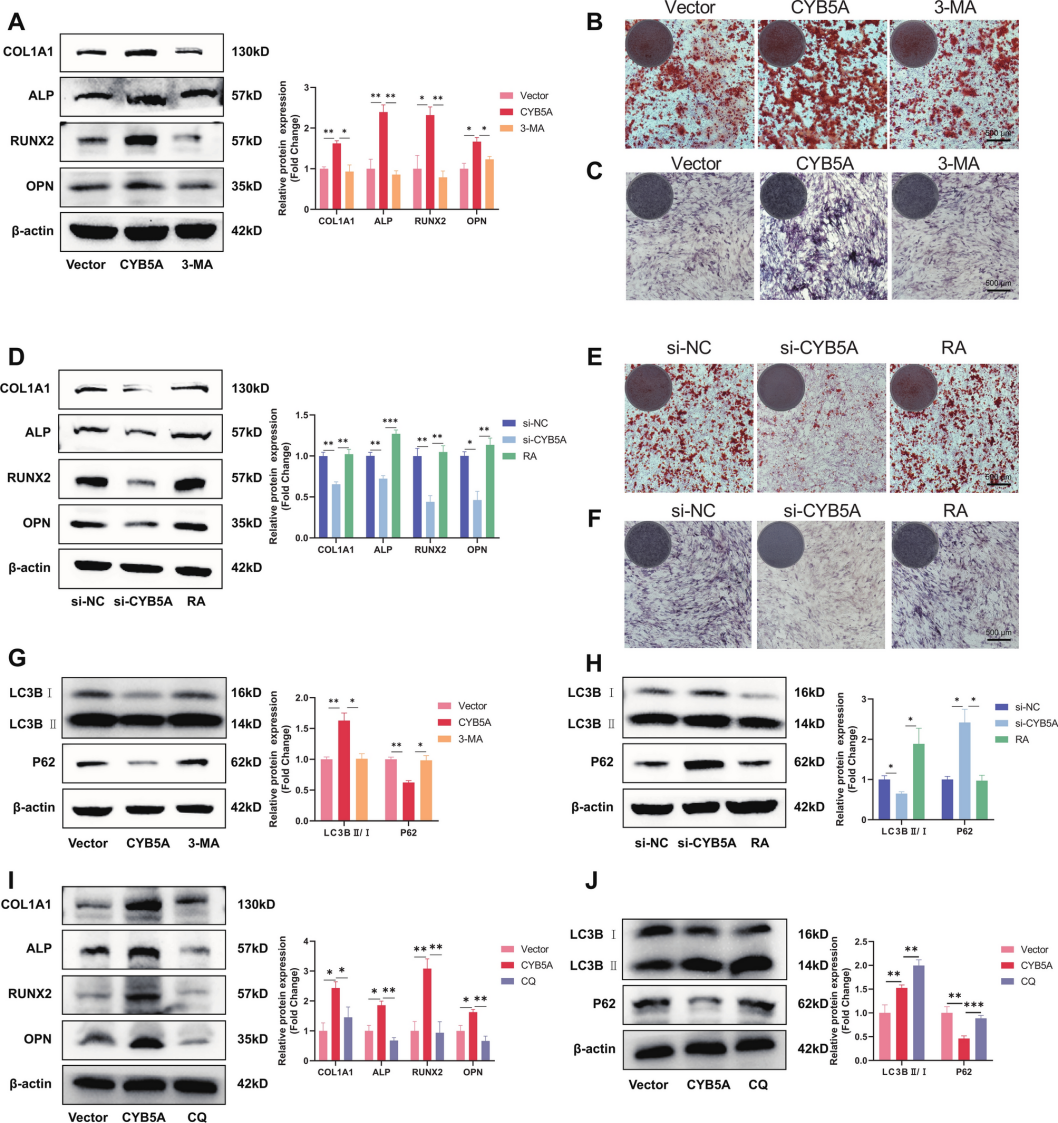
The effects of autophagy inhibitors and activators, as well as lysosomal inhibitors, on osteogenesis and autophagy in MC3T3-E1 cells. (**A**&**D**&**I**) Western Blotting and quantification showed changes in osteogenic marker (COL1A1, ALP, RUNX2, and OPN) expression. (**B**&**E**) ARS staining of MC3T3-E1 cells after osteogenic induction for 21 days (scale bar: 500 μm). (**C**&**F**) ALP staining of MC3T3-E1 cells after osteogenic induction for 14 days (scale bar: 500 μm). (**D**&**H**&**J**) Western blotting and quantification of levels of LC3-I/II and P62 in MC3T3-E1 cells. β-actin levels served as the internal normalization control. Data are presented as mean ± standard deviation (n = 3). ns = no significant difference. Statistical significance: *p < 0.05, **p < 0.01, ***p < 0.001.

The autophagy inhibitor, 3-MA, suppresses autophagosome formation^24^, whereas mTOR is inhibited by RA, an activator of cellular autophagy^25^. To examine their effects on osteogenesis and autophagy, 3-MA and RA were added to the osteogenic medium of MC3T3-E1 cells. After 3 days of osteogenic induction, COL1A1, ALP, RUNX2, and OPN expression was higher in the CYB5A group than in the Vector group; however, all these protein levels were reduced with the addition of 3-MA (Fig. 5A). Conversely, COL1A1, ALP, RUNX2, and OPN levels were lower in the si-CYB5A group than in the si-NC group; however, their levels increased following the addition of RA (Fig. 5D). After 21 days of osteogenic induction, calcium nodules were greater in the CYB5A group than in the Vector group but decreased with 3-MA addition (Fig. 5B). Calcium nodules were lower in the si-CYB5A group than in the si-NC group but increased with RA addition (Fig. 5E). Similarly, after 14 days of osteogenic induction, ALP staining was deeper in the CYB5A group than in the Vector group but became lighter with 3-MA (Fig. 5C), whereas staining was lighter in the si-CYB5A group than in the si-NC group but became deeper with RA (Fig. 5F). Furthermore, the LC3-II/LC3-I ratio was significantly higher in the CYB5A group than in the Vector group; however, it decreased after 3-MA treatment, whereas P62 protein expression showed the opposite trend (Fig. 5G). The LC3-II/LC3-I ratio in the si-CYB5A group was significantly lower than that in the si-NC group; however, it increased with RA. P62 expression was higher in the si-CYB5A group than in the si-NC group but decreased following RA treatment (Fig. 5H). Lysosomal inhibitors, CQ, block autophagic flux by preventing autophagosome-lysosome fusion^26^. After 3 days of osteogenic induction, the expression levels of COL1A1, ALP, RUNX2, and OPN were significantly upregulated in the CYB5A group; however, this upregulation was suppressed upon CQ treatment, indicating that CQ can inhibit the upregulation of osteogenic proteins (Fig. 5I). Additionally, the LC3-II/LC3-I ratio in the CYB5A group was significantly higher than that in the Vector group, but CQ treatment further increased this ration significantly. P62 expression was lower in the CYB5A group than in the Vector group but increased following CQ treatment (Fig. 5J). These findings suggest that CYB5A regulates MC3T3-E1 osteogenic differentiation via autophagy.

### CYB5A promotes osteogenic differentiation of MC3T3-E1 cells via AKT/mTOR/ULK1-mediated autophagy

**Fig. 6:**
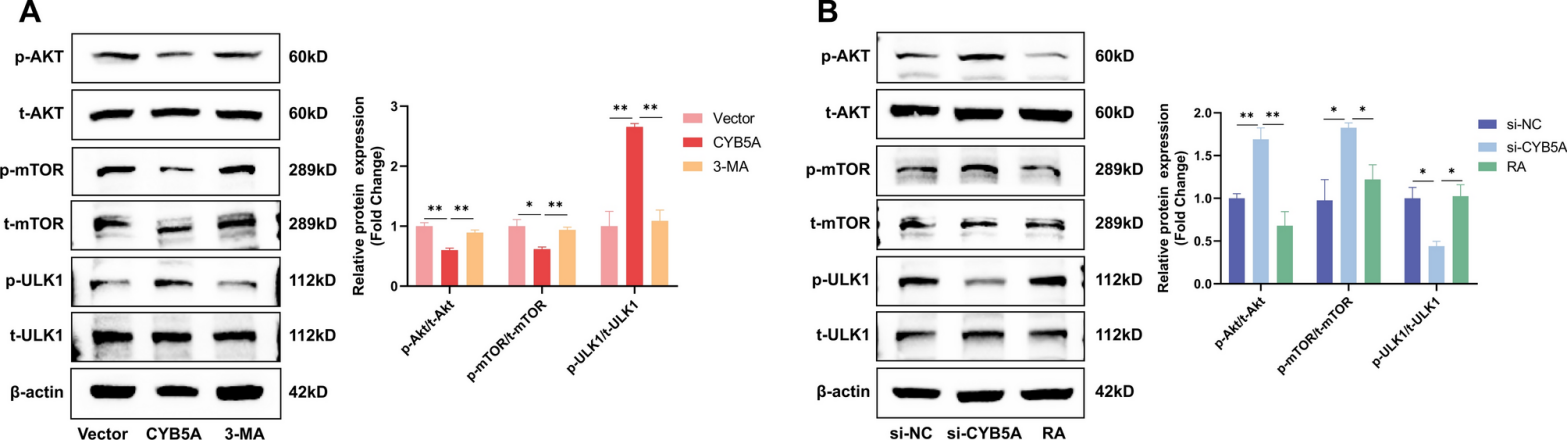
Autophagy inhibitors and activators reversed the effects of CYB5A on the AKT/mTOR/ULK1 signaling pathway. (**A**&**B**) Western Blotting and quantification assessed the levels of phosphorylated/total AKT, phosphorylated/total mTOR, and phosphorylated/total ULK1 in MC3T3-E1 cells. β-actin levels were used as the internal normalization control. Data are presented as mean ± standard deviation (n = 3). Statistical significance: *p < 0.05, **p < 0.01.

To evaluate the molecular mechanisms underlying the relationship between CYB5A-regulated osteogenic differentiation and autophagy, 3-MA and RA were used to confirm whether CYB5A regulated the osteogenic differentiation of MC3T3-E1 cells through AKT/mTOR/ULK1-mediated autophagy. The results showed that AKT and mTOR phosphorylation levels in the CYB5A group were lower than those in the Vector group, whereas the ULK1 phosphorylation level was higher. However, upon the addition of 3-MA, AKT and mTOR phosphorylation levels increased, and ULK1 phosphorylation decreased (Fig. 6A). In the si-CYB5A group, AKT and mTOR phosphorylation levels were higher than those in the si-NC group, whereas ULK1 phosphorylation was lower. The addition of RA reduced AKT and mTOR phosphorylation and increased ULK1 phosphorylation (Fig. 6B). These results suggest that CYB5A promotes osteogenic differentiation of MC3T3-E1 cells via AKT/mTOR/ULK1-mediated autophagy.

## Discussion

This study highlights the role of CYB5A in osteogenic differentiation and its regulation via autophagy. As hypothesized, CYB5A expression significantly increased during MC3T3-E1 and hMSC osteogenic differentiation, supporting its involvement in this biological process. We demonstrated that CYB5A enhanced MC3T3-E1 cell migration and differentiation without affecting their proliferation. Mechanistically, CYB5A stimulated autophagy by modulating the AKT/mTOR/ULK1 pathway, and both its overexpression and inhibition influenced osteogenic marker expression and autophagic activity. Furthermore, the use of autophagy inhibitors (3-MA) and activators (RA) confirmed that CYB5A promotes osteogenic differentiation through autophagy-dependent mechanisms. These findings indicate that CYB5A-mediated autophagy is crucial for balancing bone formation and resorption, thereby offering potential therapeutic targets for bone metabolic diseases.

The skeletal system undergoes continuous remodeling as osteoblasts form new bone and osteoclasts resorb old bone. Osteogenic differentiation involves complex signaling pathways, with several molecules promoting this process^1^. Although research into the complex molecular mechanisms underlying osteogenic differentiation is ongoing, the goal is to develop new strategies for preventing and treating bone metabolism-related diseases. Bioinformatics technology has become a powerful tool for exploring the molecular mechanisms of osteogenic differentiation and has provided new insights into diagnosing and treating bone metabolic disorders^27^. MC3T3-E1 cells are widely used as a model for osteogenesis research and to investigate the roles of specific genes, proteins, and signaling pathways involved in bone formation^28^. In this study, bioinformatics analysis of gene expression profiling data (GSE30393, GSE46400, GSE37558, and GSE80614) revealed CYB5A upregulation during MC3T3-E1 and hMSC osteogenic differentiation. Therefore, we hypothesized that CYB5A plays a significant role in osteogenic differentiation.

Genetic engineering has been widely applied in osteoblast research using various gene vectors to introduce exogenous genes into osteoblasts, thereby enhancing their differentiation potential and promoting bone regeneration and repair^29^. Plasmids and siRNAs are essential tools in genetic engineering and are critical for both gene therapy and functional gene studies^27,30^. In this study, we used plasmids to overexpress CYB5A in MC3T3-E1 cells and siRNAs to silence CYB5A expression. During osteogenic differentiation, osteoblasts are regulated by various signaling molecules and complex molecular pathways that control proliferation and migration, thus establishing favorable conditions for differentiation^31^. CCK-8 and EdU assays indicated that CYB5A did not significantly affect proliferation. However, migration analysis using scratch and Transwell assays revealed that CYB5A overexpression promoted cell migration, whereas knockdown impaired it. Therefore, we propose that CYB5A positively regulates MC3T3-E1 cell migration.

To date, the role of CYB5A in osteogenic differentiation has received limited attention. CYB5A expression was significantly down-regulated when osteoblasts were exposed to adverse endogenous or exogenous stimuli, resulting in diminished osteogenic differentiation potential^32,33^. This study revealed that CYB5A overexpression significantly increased the expression of osteogenic markers (COL1A1, ALP, RUNX2, and OPN) at both the mRNA and protein levels, enhanced the formation of calcium nodules, and intensified ALP staining in MC3T3-E1 cells. Conversely, CYB5A knockdown reduced the expression of osteogenic markers, decreased calcium nodule formation, and weakened ALP staining. These findings demonstrate that CYB5A positively regulates the osteogenic differentiation of MC3T3-E1 cells.

Autophagy plays a pivotal role in the pathogenesis and progression of skeletal disorders^34^. Autophagy is essential for maintaining cell viability, and the regulation of autophagic processes has significant therapeutic potential for preventing and treating bone metabolic disorders^4^. Suppressing ATG protein expression reduces bone mass, whereas enhanced autophagy promotes osteogenic differentiation^35^. CYB5A participates in regulating cellular activities through autophagy^18^. During autophagosome biogenesis, LC3-I is modified and processed into its lipidated variant, LC3-II^36^. P62 mediates the degradation of ubiquitinated proteins or other substrates. However, P62 accumulation impairs autophagic degradation, leading to autophagy inhibition^37^. In this study, we found that CYB5A overexpression increased the LC3-II/LC3-I ratio and promoted P62 protein degradation, whereas CYB5A knockdown decreased the ratio and reduced P62 degradation. These findings suggest that CYB5A positively regulates autophagy in MC3T3-E1 cells. The migration of osteoblasts to the site of bone formation plays an indispensable role in bone development, remodeling, and healing processes^38^. Chemical, mechanical, growth, and other factors can enhance cell migration and improve bone formation and remodeling^39,40^. Autophagy is an essential regulator of cell migration, and a decrease in autophagy function leads to a reduction in cell migration^41^. In this study, CYB5A positively regulated autophagy in MC3T3-E1 cells while also promoting cell migration. This demonstrates that CYB5A promotes the migration of MC3T3-E1 cells by enhancing autophagy.

Osteogenic differentiation is governed by a complex network of signaling pathways that regulate cell fate and bone formation in a coordinated manner^1^. Activation of the Notch signaling pathway stimulates the proliferation of osteoprogenitor cells while inhibiting their differentiation into mature osteoblasts by suppressing Wnt signaling^42^. In contrast, activation of the Wnt and BMP signaling pathways accelerates the maturation of osteoblasts, promoting new bone formation^43^. Autophagy is essential for osteogenic differentiation. Activation of mTOR-dependent autophagy through pharmacological interventions or genetic manipulations enhances osteogenic differentiation and promotes new bone formation^44,45^. ULK1, a key autophagy initiator, is modulated by multiple upstream signaling pathways^9^, with the AKT/mTOR pathway playing a pivotal role in autophagy regulation by inhibiting ULK1 activation^46^. Both ERK1/2 and ULK1 are essential signaling molecules that regulate various cellular processes, and the activation of ERK1/2 influences ULK1 activity^47^. Previous studies reported that increased CYB5A expression reduces ERK1/2 and AKT phosphorylation^48^. In this study, elevated CYB5A levels decreased AKT and mTOR phosphorylation, thereby activating ULK1 and promoting autophagy. Conversely, CYB5A knockdown increased AKT and mTOR phosphorylation, inhibiting ULK1 and reducing autophagy. Our findings also indicate that neither CYB5A overexpression nor knockdown affected ERK1/2 protein levels, suggesting that CYB5A’s modulation of ERK1/2 may depend on the cell type. The effects of CYB5A overexpression or knockdown on osteogenic differentiation, autophagy, and the AKT/mTOR/ULK1 pathway in MC3T3-E1 cells were reversed by the autophagy inhibitor (3-MA) or activator (RA). In CYB5A-overexpressing MC3T3-E1 cells, the lysosomal inhibitor (CQ) blocked autophagic flux, concomitant with downregulated expression of osteogenic-related proteins. Collectively, these findings demonstrate that CYB5A regulates the osteogenic differentiation of MC3T3-E1 cells through autophagy mediated by the AKT/mTOR/ULK1 signaling pathway. Thus, CYB5A is a potential target for modulating osteogenic differentiation and treating bone metabolism disorders.

Despite these promising results, one limitation of this study is that MC3T3-E1 cells, widely used in osteoblast research, differ from primary cultured osteoblasts, limiting their clinical relevance. Future studies should confirm the role of CYB5A in primary cultured osteoblasts and investigate whether these findings are true in vivo. In addition, MC3T3-E1 cells are cultured in monolayers, which fail to replicate in vivo bone formation. Exploring the function of CYB5A in other skeletal cells, such as osteoclasts, osteocytes, and chondrocytes, would deepen the understanding of its role in bone metabolism. Further research should examine how CYB5A functions under different physiological and pathological conditions, such as osteoporosis and bone fracture healing, to assess its therapeutic potential.

In summary, this study demonstrated that CYB5A is upregulated during the osteogenic differentiation of MC3T3-E1 cells and positively regulates this process via autophagy mediated by the AKT/mTOR/ULK1 signaling pathway. Therefore, CYB5A may serve as a potential therapeutic target for preventing and treating bone metabolic diseases. Further investigations are required to explore its role in vivo and assess its potential for clinical applications in bone repair and regeneration.

## Supplementary Information

Supplementary Information.

## Data Availability

The datasets extracted and analyzed during the current study are available in the Gene Expression Omnibus repository, [GSE30393] (https://www.ncbi.nlm.nih.gov/geo/query/acc.cgi?acc=GSE30393), [GSE46400] (https://www.ncbi.nlm.nih.gov/geo/query/acc.cgi?acc=GSE46400), [GSE37558] (https://www.ncbi.nlm.nih.gov/geo/query/acc.cgi?acc=GSE37558), and [GSE80614] (https://www.ncbi.nlm.nih.gov/geo/query/acc.cgi?acc=GSE80614).
