# Supplementary material for: CYB5A promotes osteogenic differentiation of MC3T3-E1 cells through autophagy mediated by the AKT/mTOR/ULK1 signaling pathway
Source: Sci Rep. 2025 Apr 17;15:13234. doi: 10.1038/s41598-025-97086-0 (PMC12006315; doi:10.1038/s41598-025-97086-0)
Supplement: Supplementary file 1 — Supplementary Information. [file 41598_2025_97086_MOESM1_ESM.pdf]

Supplementary Figure for:

CYB5A promotes osteogenic differentiation of MC3T3-E1 cells through autophagy mediated by the AKT/mTOR/ULK1 signaling pathway

Yanjie Zhang[1,2†], Jinmeng Li[1,2†], Beibei Liu[1,2], Peilin Wang[2,3],
Hanyu Xiao[1,2], Qingfu Wang[1,2], Ruixin Li[2], Jian Zhang[1,2*]

1. Department of Oral Implantology, Tianjin Stomatological Hospital, School of Medicine, Nankai University, Tianjin 300041, China
2. Tianjin Key Laboratory of Oral and Maxillofacial Function Reconstruction, Tianjin 300041, China
3. Department of Oral Mucosal Diseases, Tianjin Stomatological Hospital, School of Medicine, Nankai University, Tianjin 300041, China

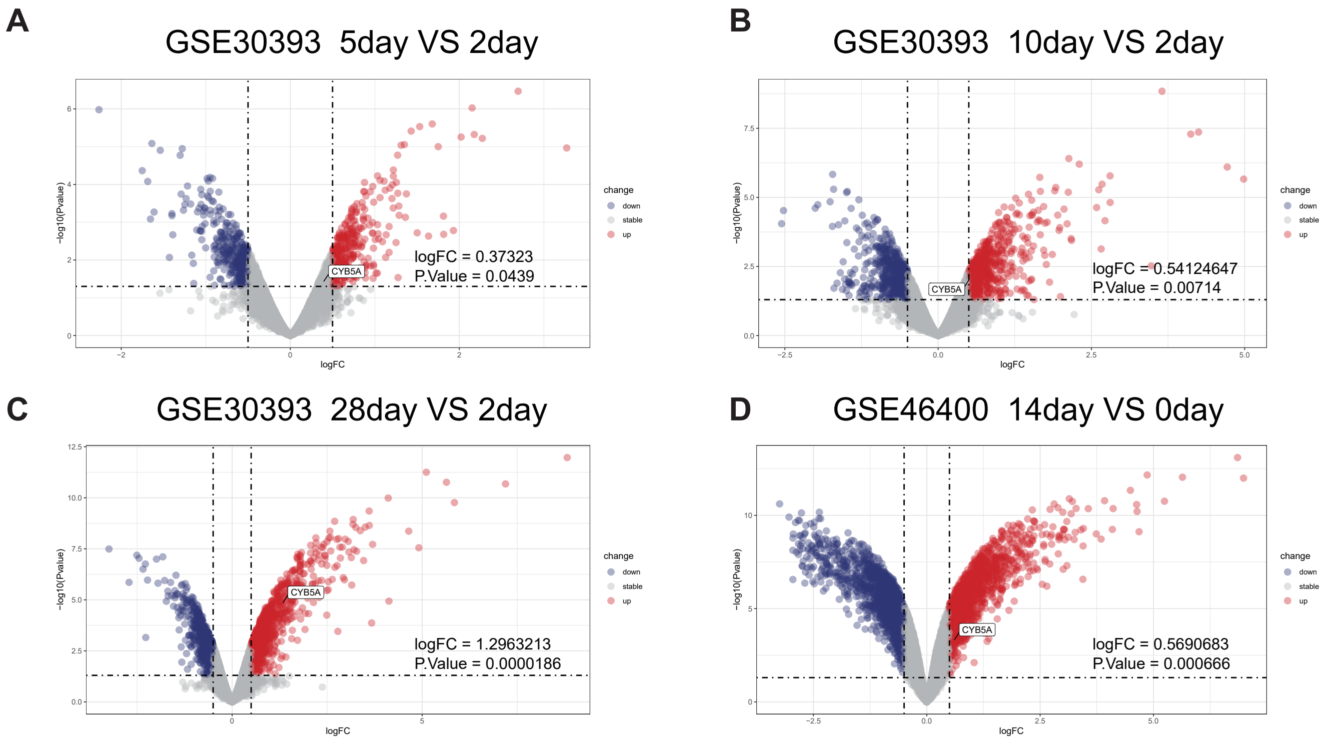

Supplementary Figure 1. Gene expression profiling data were obtained from the GEO database and analyzed for differential expression. (A) Differences between 5 and 2 days of GSE30393 osteogenic induction. (B) Differences between 10 and 2 days of GSE30393 osteogenic induction. (C) Differences between 28 and 2 days of GSE30393 osteogenic induction. (D) Differences between 14 and 0 days of GSE46400 osteogenic induction.

Dataset: GSE30393
Gene expression analysis of murine MC3T3-E1 cells induced to undergo synchronized osteoblastic differentiation in vitro
Osteogenic differentiation time and sample size: 2day(3), 5day(3), 10day(3), 28day(3)
Contributors: Dar M, Gesty-Palmer D, El-Shewy HM, Luttrell LM
Dataset Link: https://www.ncbi.nlm.nih.gov/geo/query/acc.cgi?acc=GSE30393

Dataset: GSE46400
Comparison of gene expression profiles between MC3T3-E1 without osteoblastic induction and with 14 days of ostoblastic induction
Osteogenic differentiation time and sample size: 0day(3), 14day(3)
Contributors: He Q, Swindle CS, Wan C, Flynn RJ, Chen D, Klug CA
Dataset Link: https://www.ncbi.nlm.nih.gov/geo/query/acc.cgi?acc=GSE46400

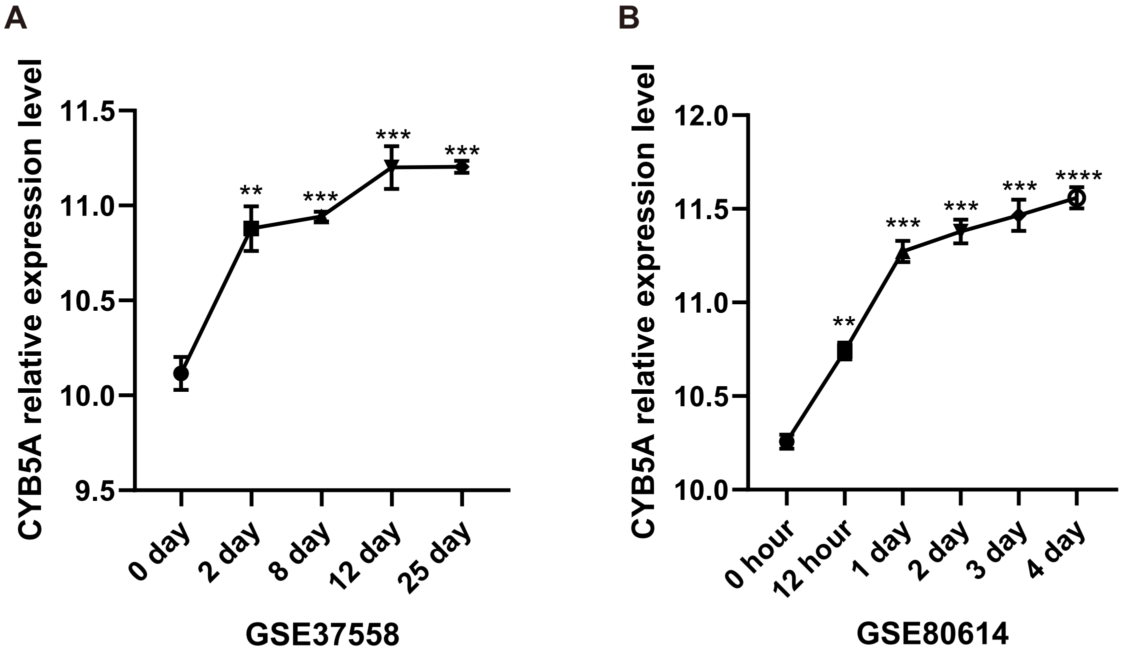

Supplementary Figure 2. Gene expression profiling data were obtained from the GEO database and analyzed for differential expression. (A) Differential expression of CYB5A in hMSCs after osteogenic induction in GSE37558. (B) Differential expression of CYB5A in hMSCs after osteogenic induction in GSE80614. Statistical significance: $^{**}p < 0.01$, $^{***}p < 0.001$, $^{****}p < 0.0001$.

Dataset: GSE37558
Calcifying vascular smooth muscle cells and osteoblasts: independent cell types exhibiting extracellular matrix and biomineralization-related mimicries.
Osteogenic differentiation time and sample size: 0day(4), 2day(3), 8day(3), 12day(3), 25day(3)
Contributors: Rodrigo A, Eijken M, van de Peppel J, van Leeuwen JP
Dataset Link: https://www.ncbi.nlm.nih.gov/geo/query/acc.cgi?acc=GSE37558

Dataset: GSE80614
Gene expression analyses of the differentiating hMSC into Osteoblasts and Adipocytes
Osteogenic differentiation time and sample size: 0hour(3), 12 hour(3), 1day(3), 2day(3), 3day(3), 4day(3)
Contributors: van de Peppel J, Strini T, Tilburg J, Westerhoff H, van Wijnen AJ, van Leeuwen JP
Dataset Link: https://www.ncbi.nlm.nih.gov/geo/query/acc.cgi?acc=GSE80614

Gene	Forward primer (5′→3′)	Reverse primer (3′→5′)
CYB5A	GAGCATCCTGGTGGAGAAGA	TCTCGTGCATCCGTAGAGTG
RUNX2	CCGAAATGCCTCCGCTGTTATG	GGATTTGTGAAGACTGTTATGGT
ALP	TATGTCTGGAACCGCACTGAAC	CACTAGCAAGAAGAAGCCTTTGG
COL1A1	CCTGAGCCAGCAGATTGA	TCCGCTCTTCCAGTCAG
OPN	AGCAAGAAACTCTTCCAAGCAA	GTGAGATTCGTCAGATTCATCCG
β-actin	CATCCGTAAAGACCTCTATGCCAAC	ATGGAGCCACCGATCCACA

Supplementary Table 1. Primer sequences used for qRT-PCR.

Reagents	CAT number	Company	Proportions
CYB5A	2365-1-AP	Proteintech	1/500
COL1A1	#39952	Cell Signaling Technology	1/1000
RUNX2	#12556	Cell Signaling Technology	1/1000
ALP	A0514	ABclonal	1/1000
OPN	80912-4-RR	Proteintech	1/1000
LC3 Ⅰ/Ⅱ	WL01506	Wanleibio	1/1000
P62	#5114	Cell Signaling Technology	1/1000
p-ERK	WLP1512	Wanleibio	1/1000
t-ERK	WL01864	Wanleibio	1/1000
p-AKT	WLP001a	Wanleibio	1/1000
t-AKT	WL0003b	Wanleibio	1/1000
p-mTOR	WL03694	Wanleibio	1/1000
t-mTOR	WL02477	Wanleibio	1/1000
p-ULK	AP1495	ABclonal	1/2000
t-ULK	WL03067	Wanleibio	1/1000
β-actin	81115-1-RR	Proteintech	1/5000
HRP-conjugated Goat Anti-Rabbit IgG(H+L)	RGAR001	Proteintech	1/5000

Supplementary Table 2. Antibody used for western blotting.

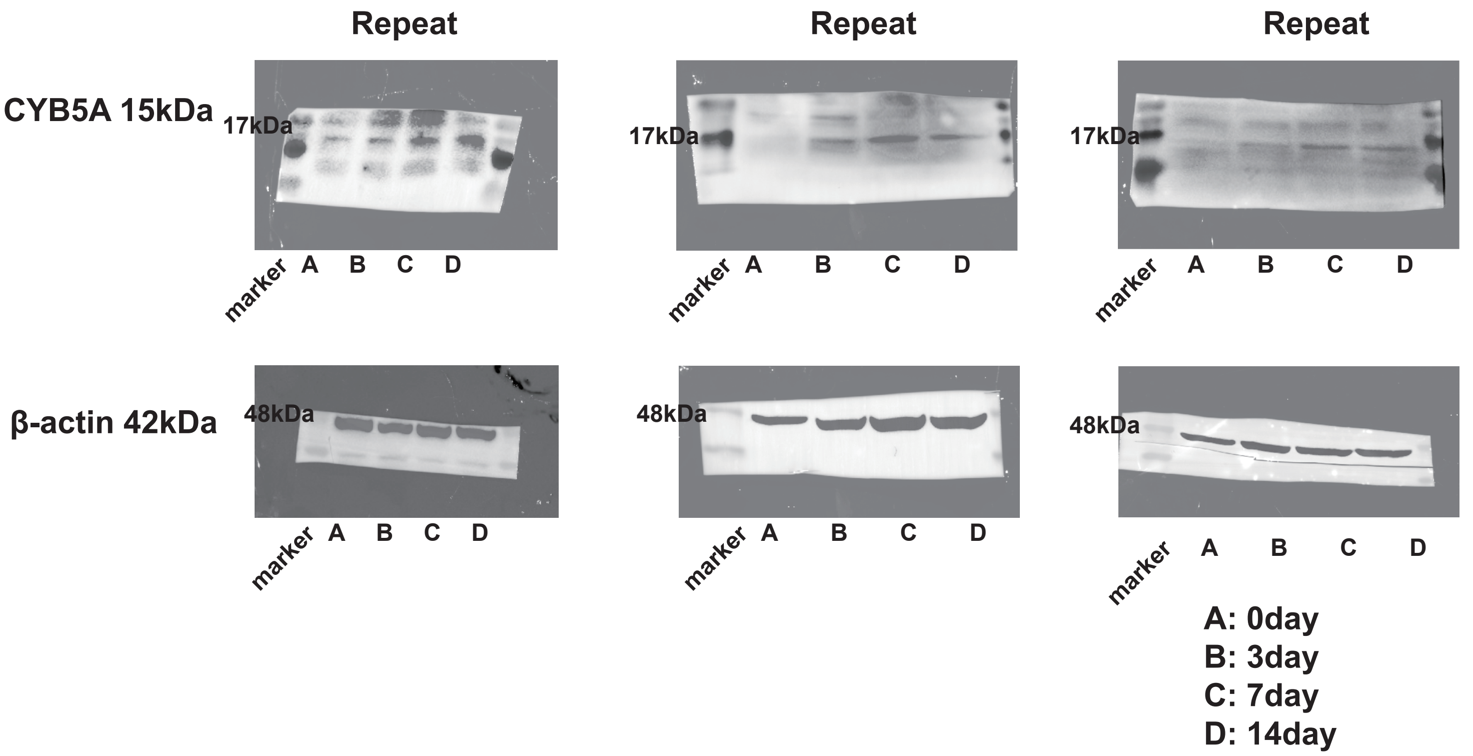

Supplementary Figure 3. Unprocessed Western blotting. Source Data for Figure 1B.

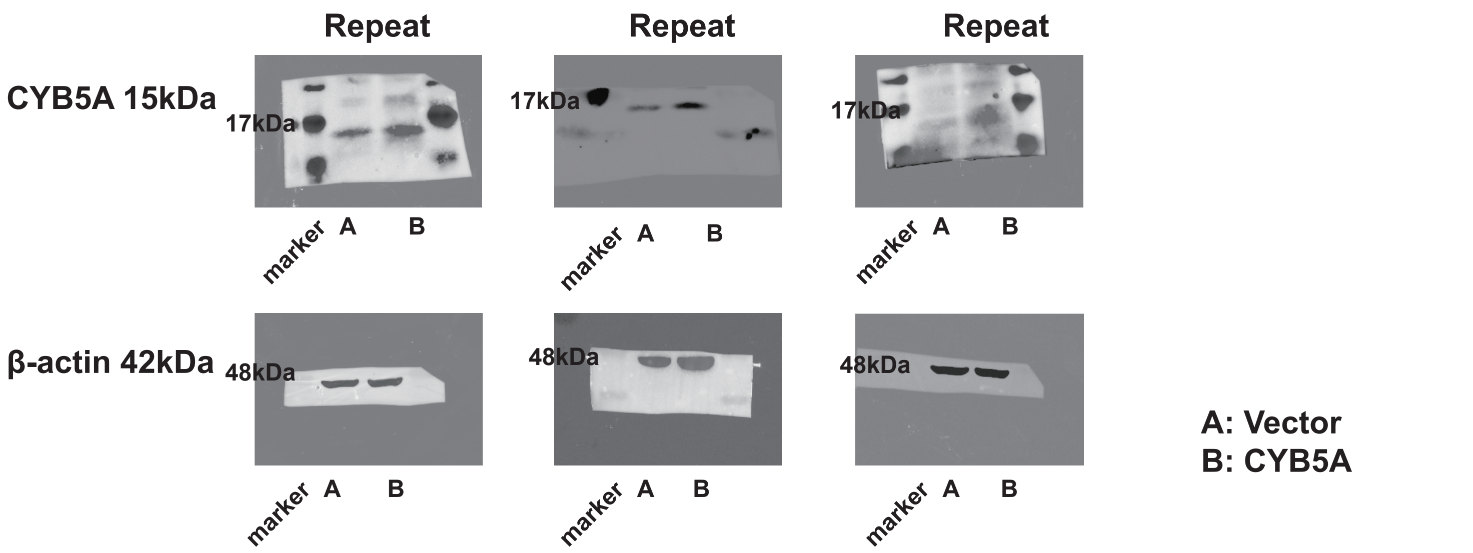

Supplementary Figure 4. Unprocessed Western blotting. Source Data for Figure 2B.

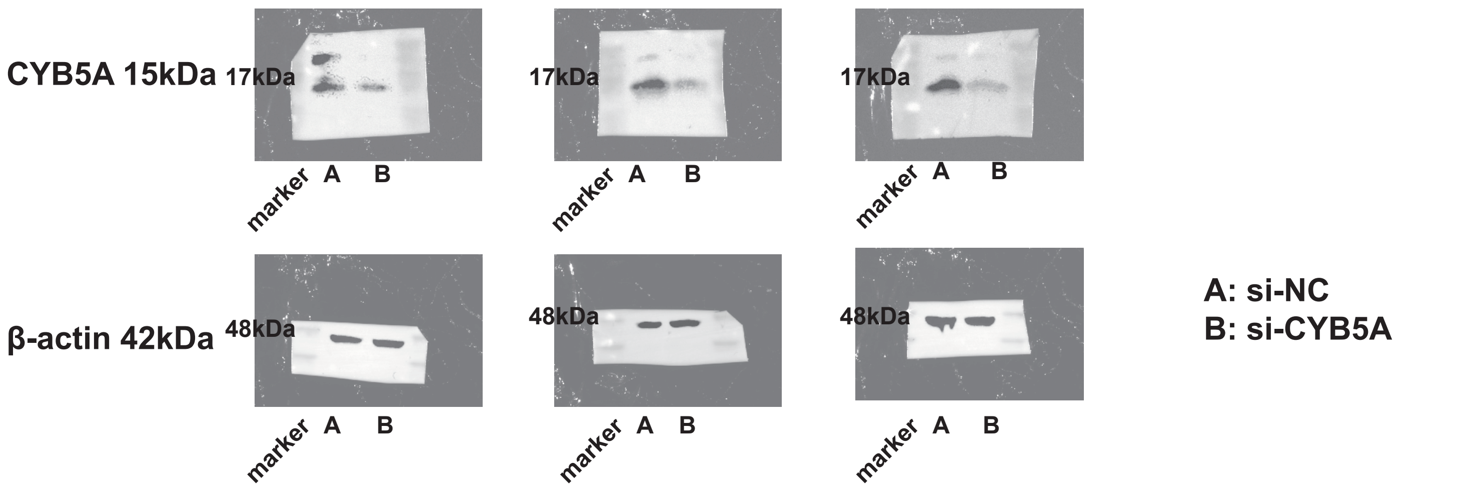

Supplementary Figure 5. Unprocessed Western blotting. Source Data for Figure 2H.

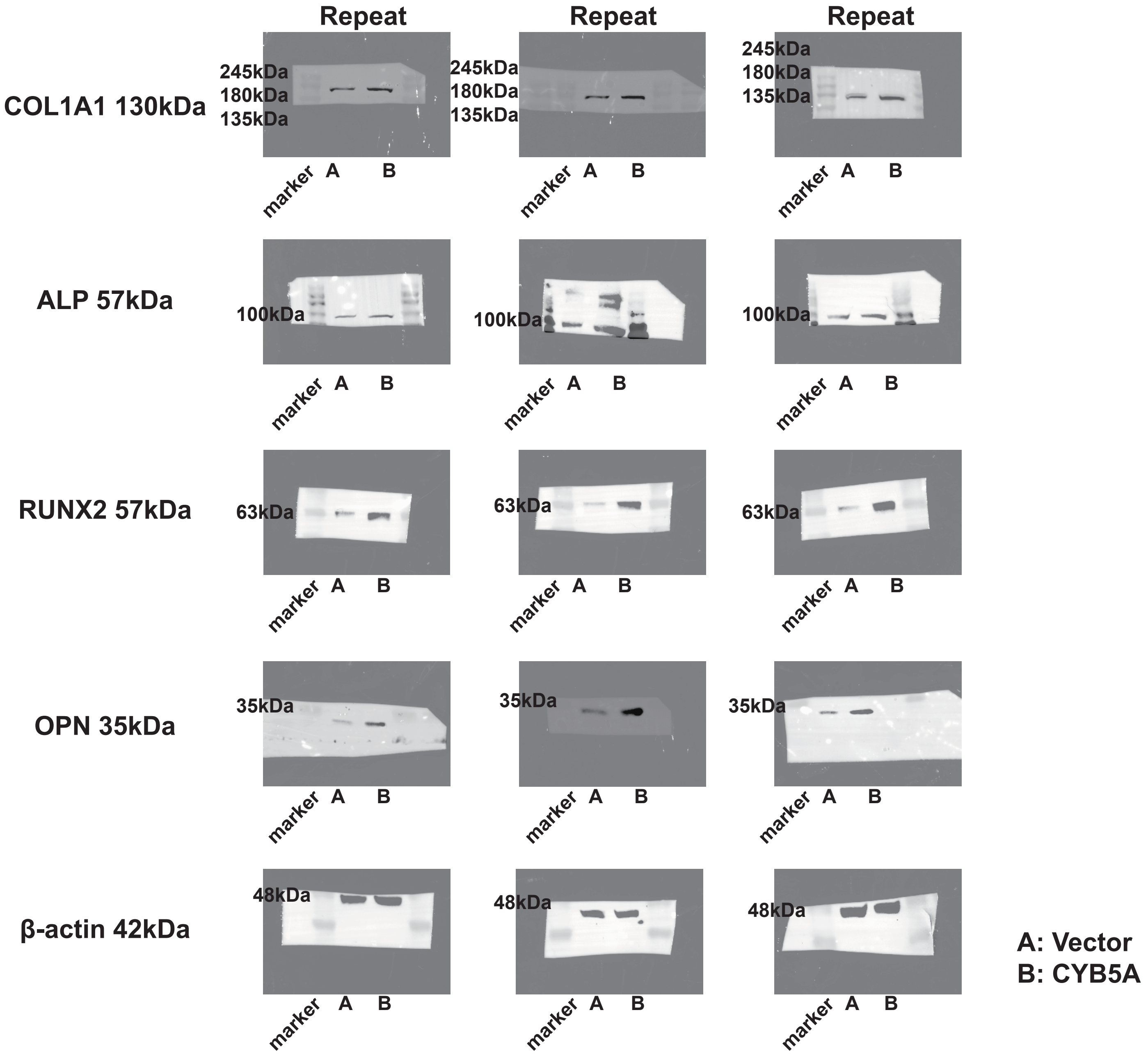

The theoretical molecular weight of ALP is 57kDa,
but the actual observed molecular weight is around 80kDa.

Supplementary Figure 6. Unprocessed Western blotting. Source Data for Figure 3B (3day).

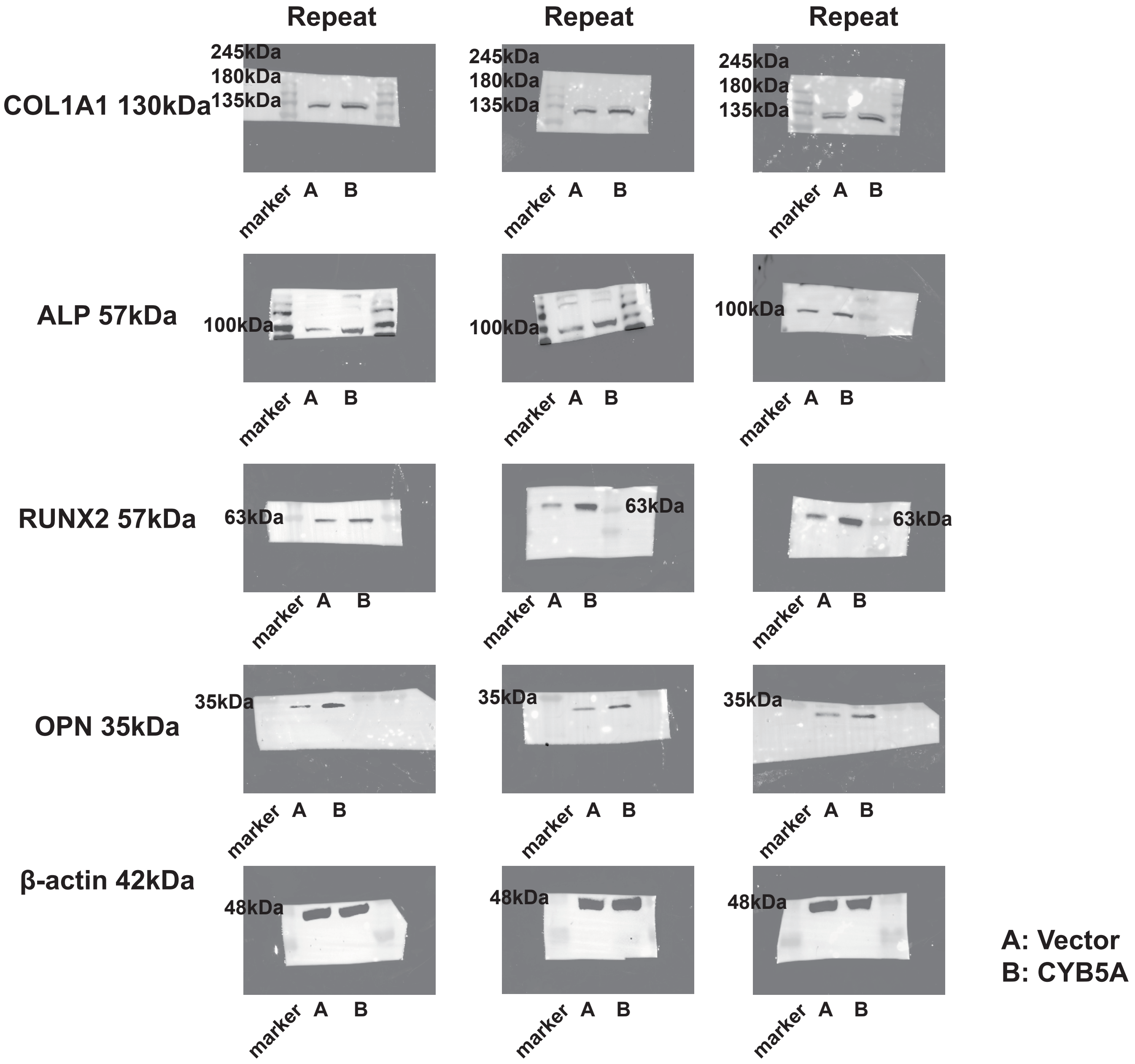

Supplementary Figure 7. Unprocessed Western blotting. Source Data for Figure 3B (7day).

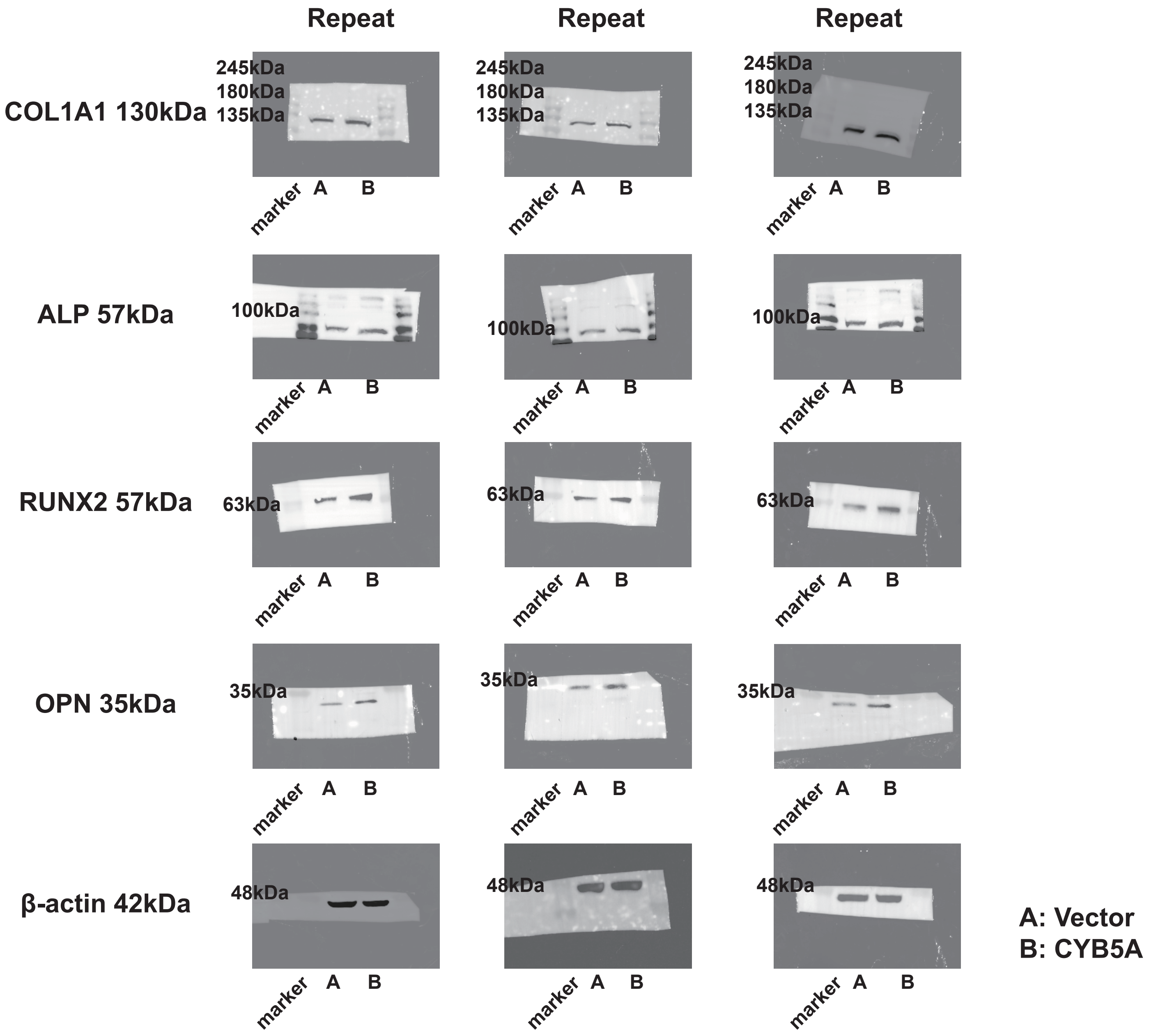

Supplementary Figure 8. Unprocessed Western blotting. Source Data for Figure 3B (14day).

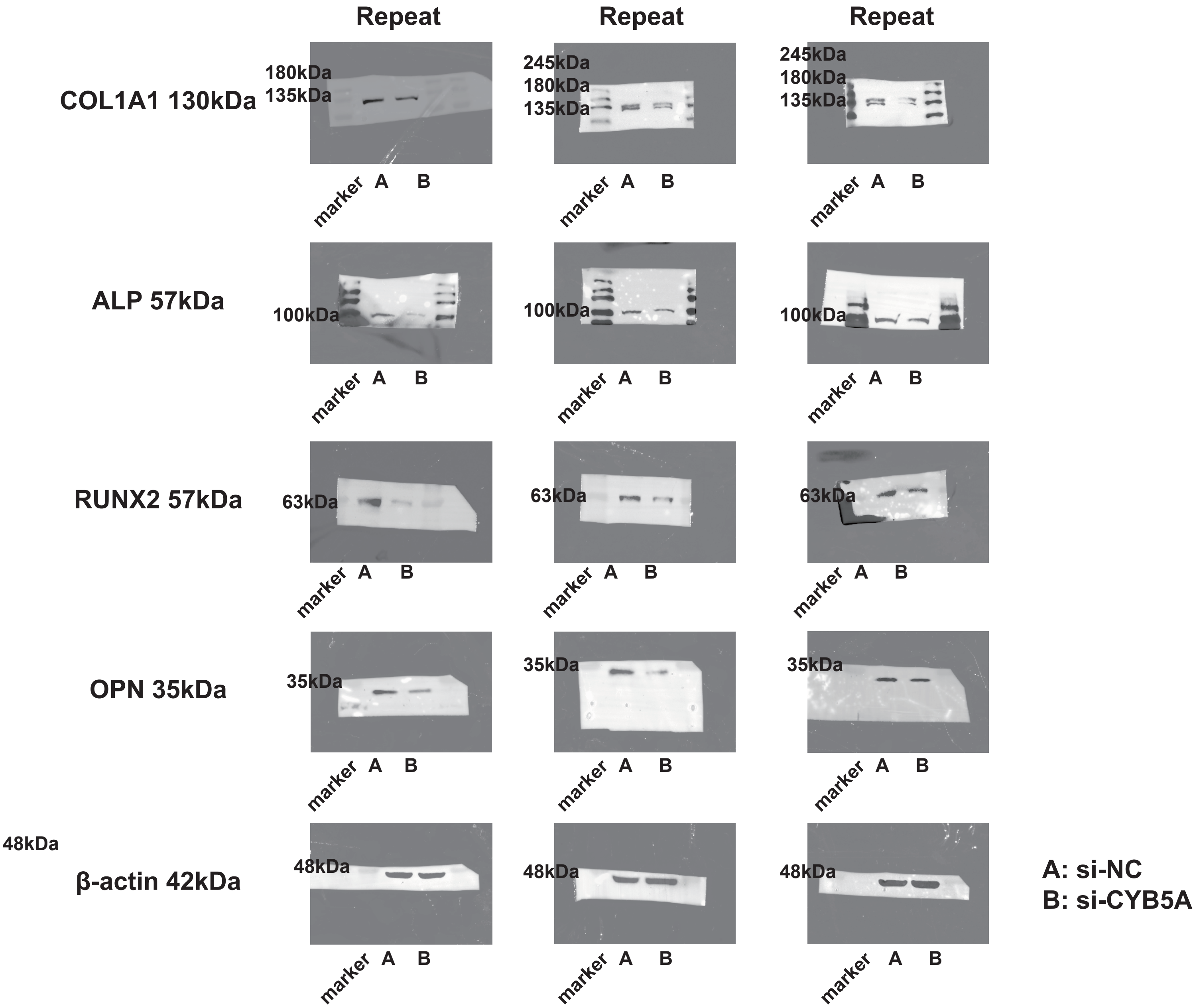

Supplementary Figure 9. Unprocessed Western blotting. Source Data for Figure 3F (3day).

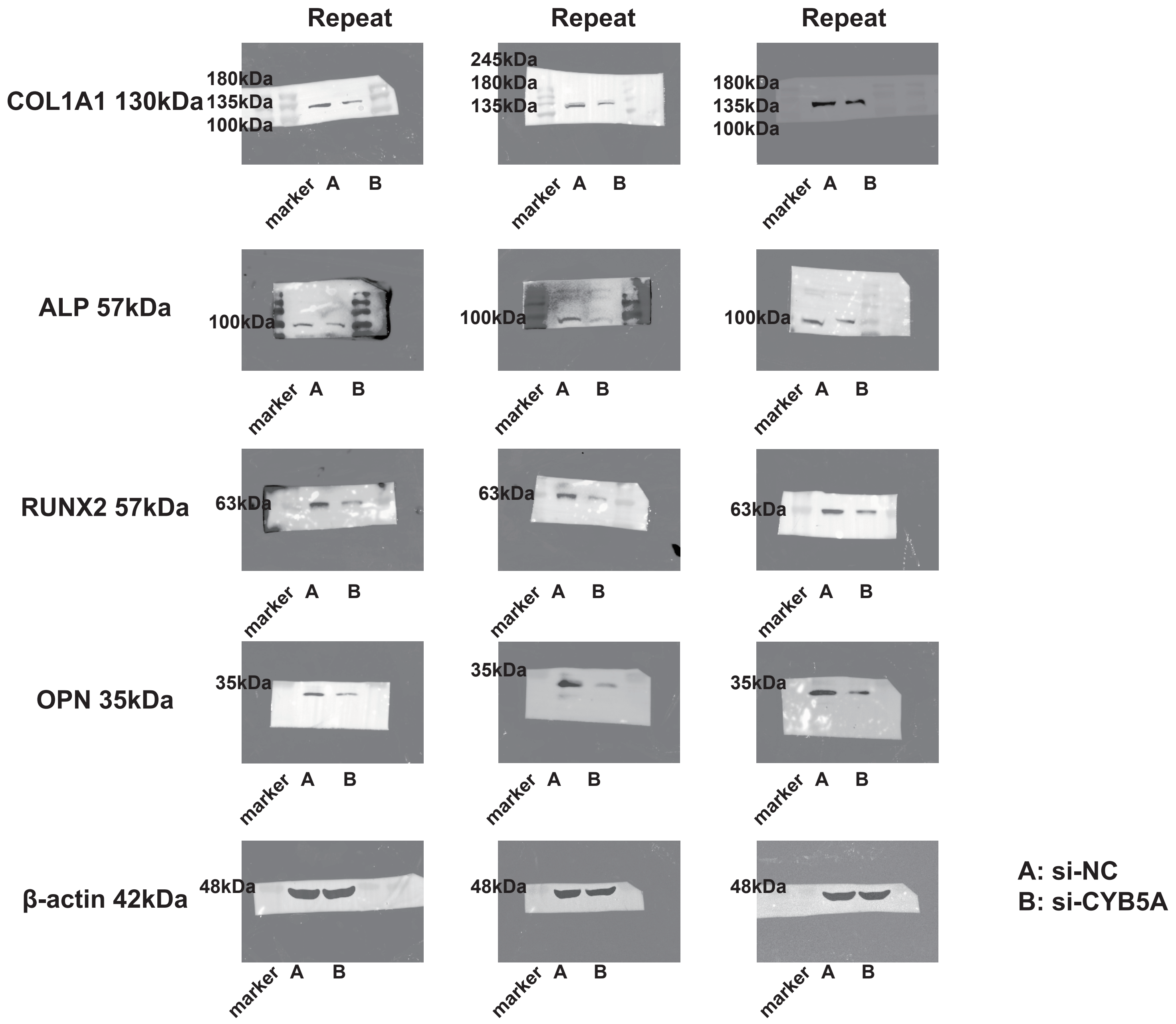

Supplementary Figure 10. Unprocessed Western blotting. Source Data for Figure 3F (7day).

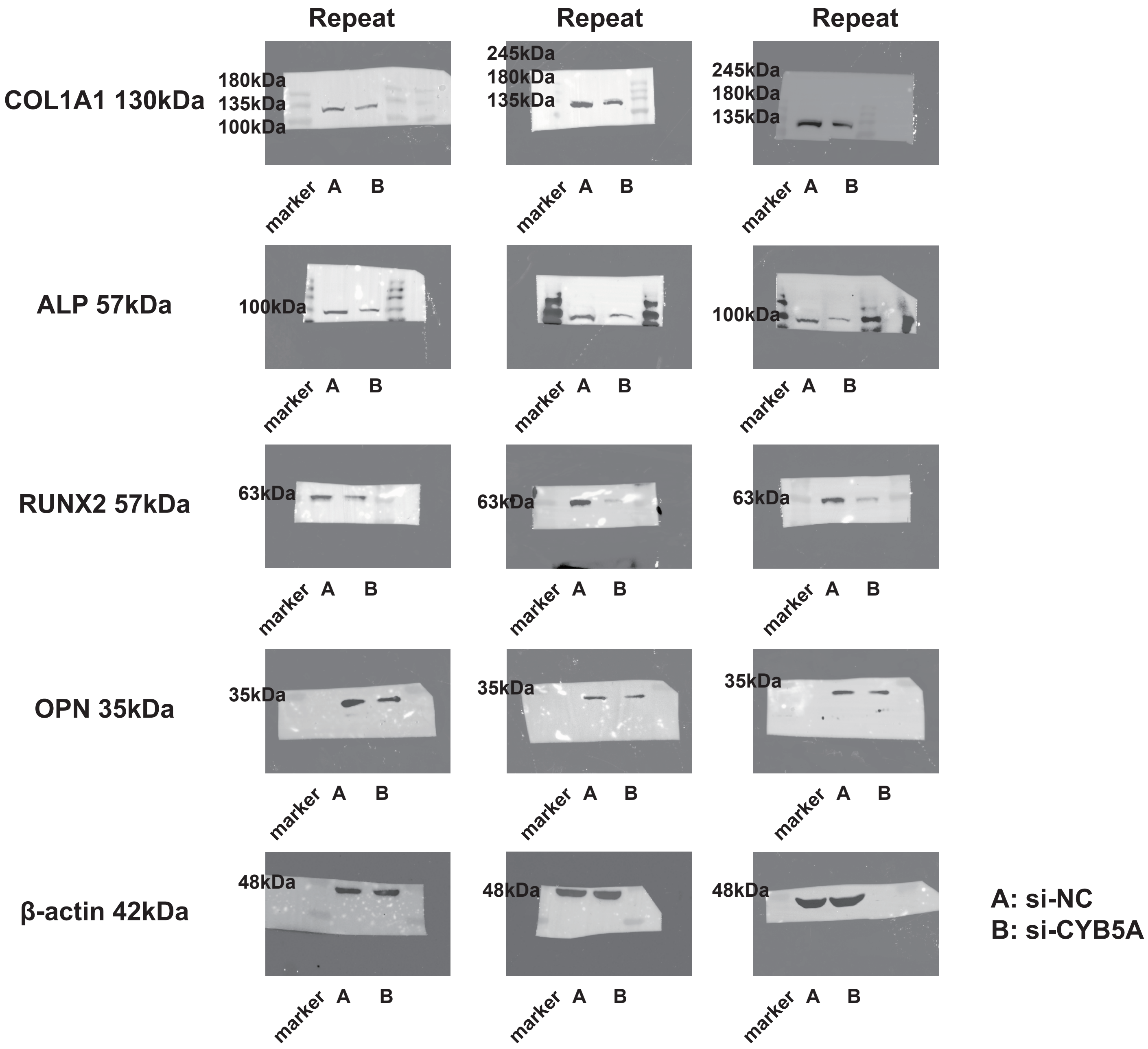

Supplementary Figure 11. Unprocessed Western blotting. Source Data for Figure 3F (14day).

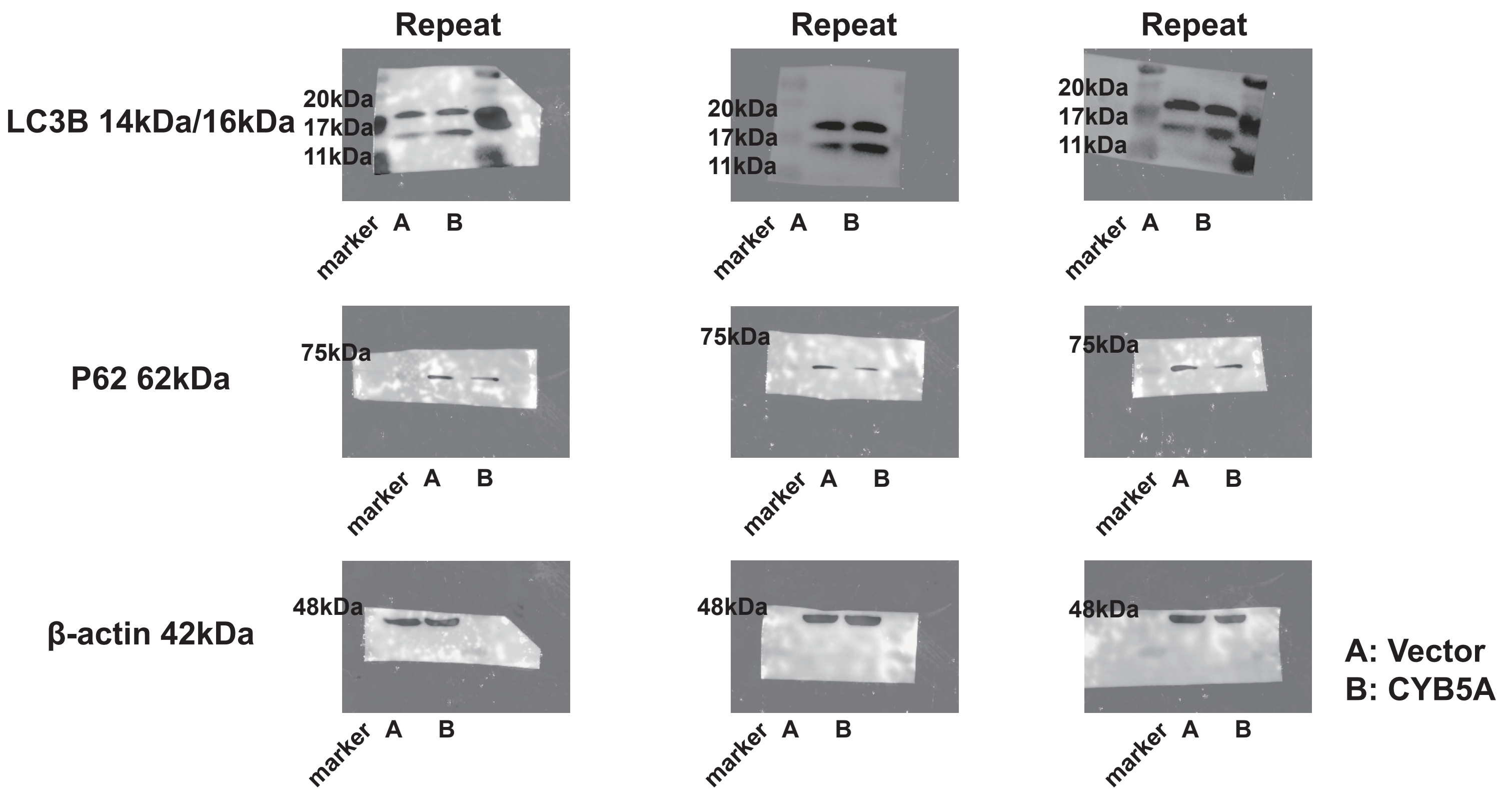

Supplementary Figure 12. Unprocessed Western blotting. Source Data for Figure 4A (3day).

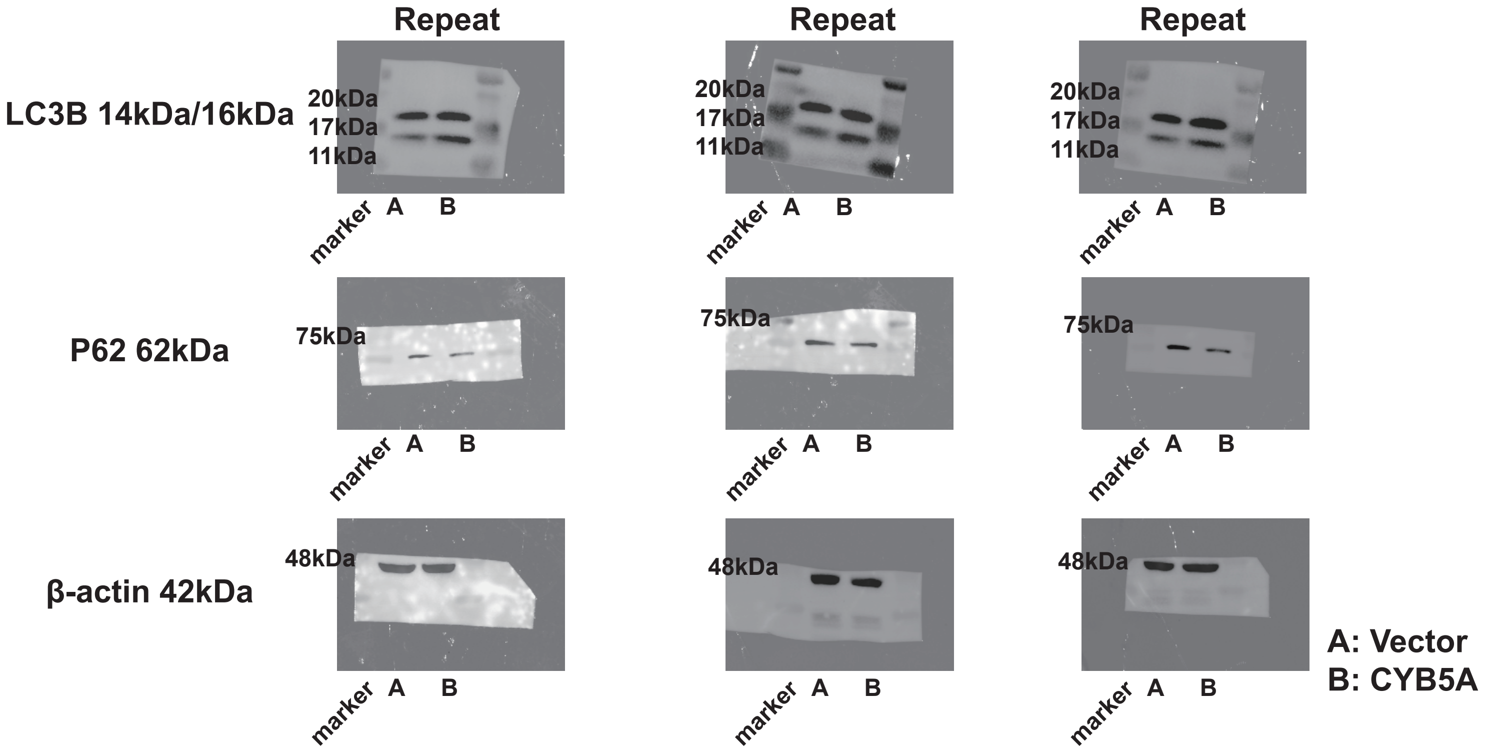

Supplementary Figure 13. Unprocessed Western blotting. Source Data for Figure 4A (7day).

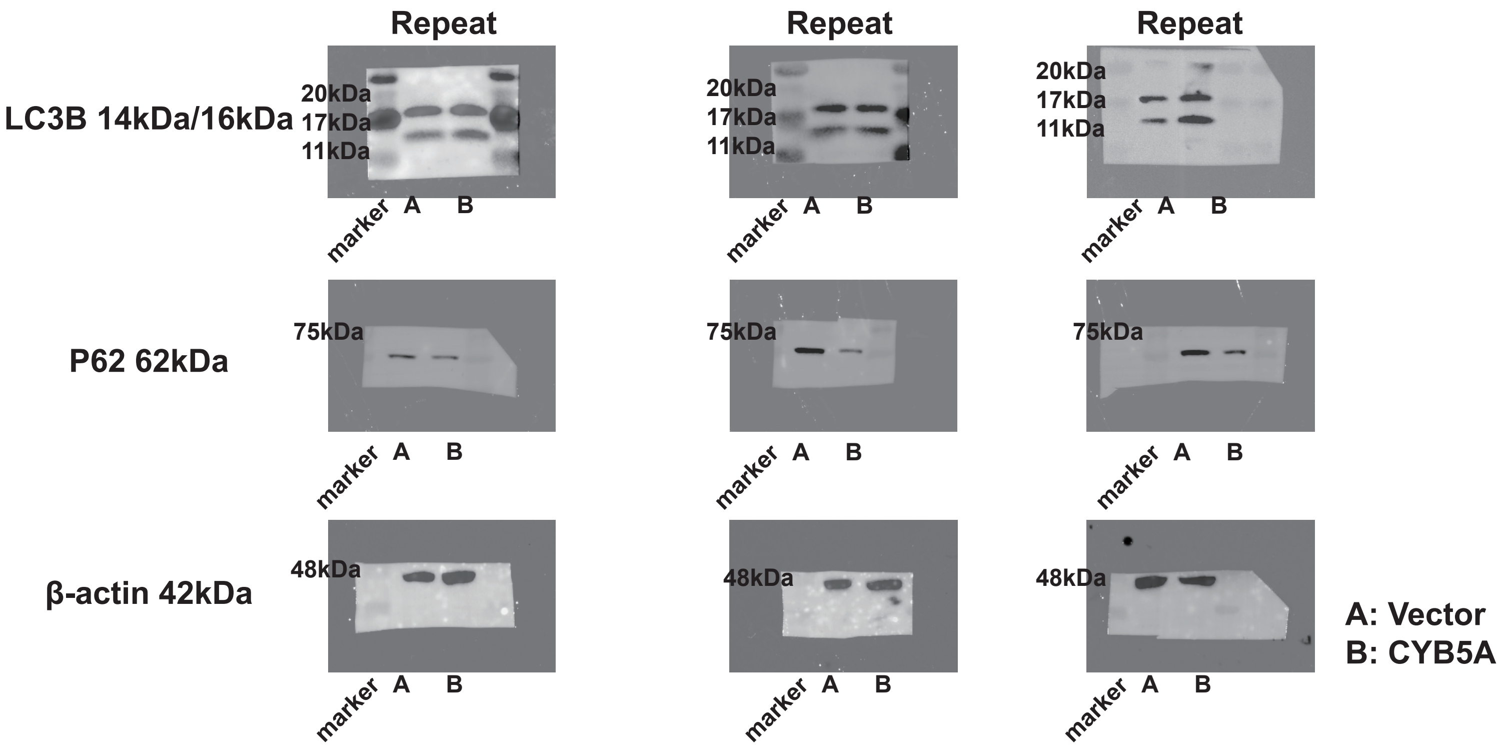

Supplementary Figure 14. Unprocessed Western blotting. Source Data for Figure 4A (14day).

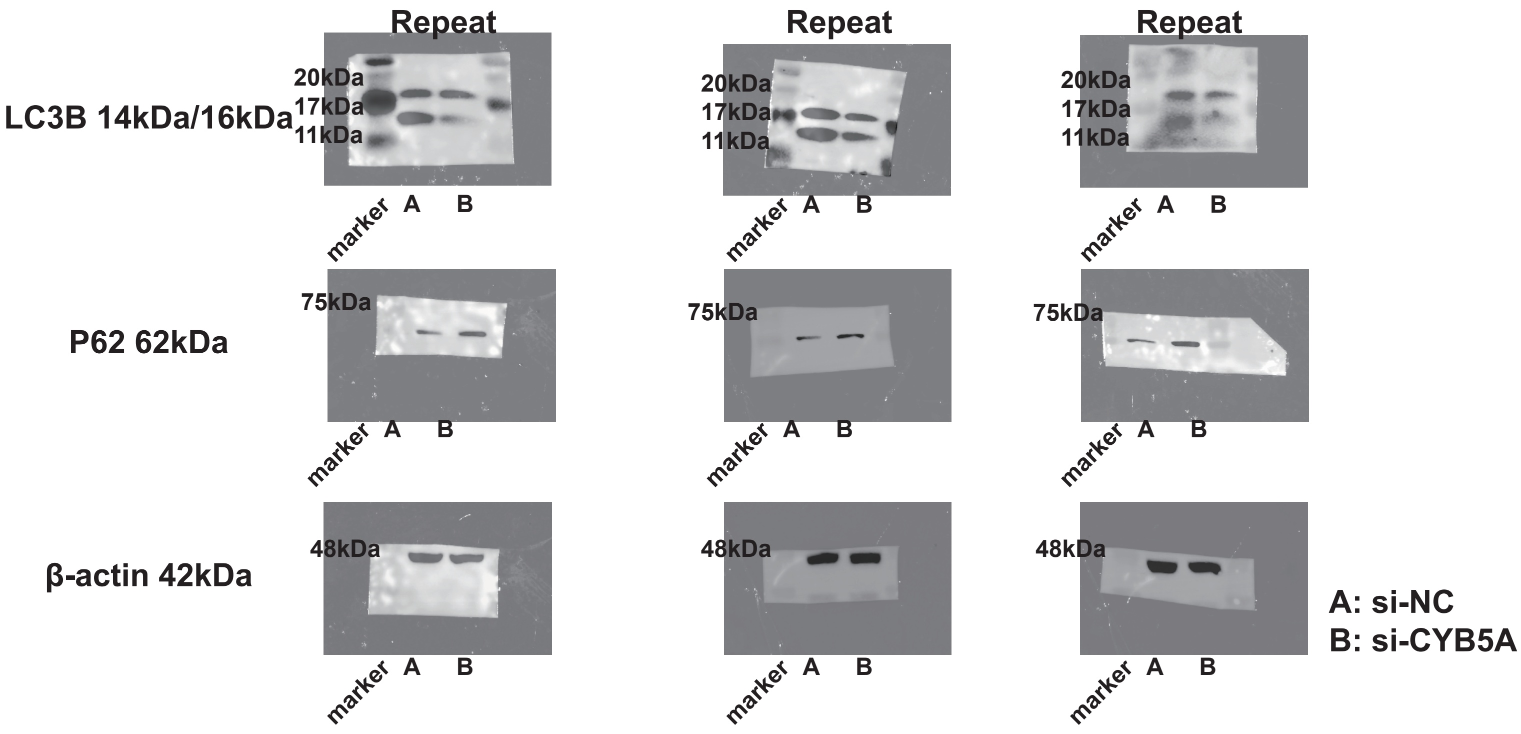

Supplementary Figure 15. Unprocessed Western blotting. Source Data for Figure 4B (3day).

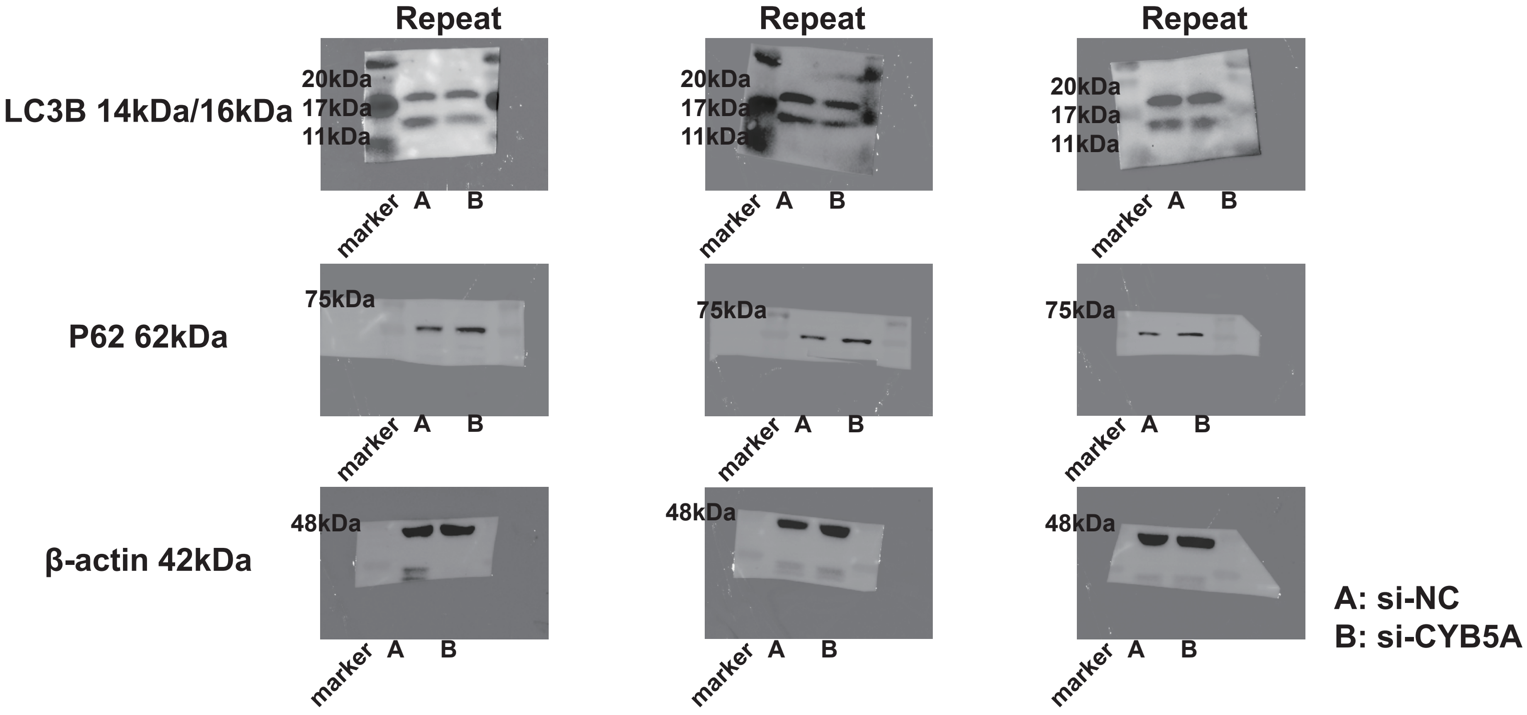

Supplementary Figure 16. Unprocessed Western blotting. Source Data for Figure 4B (7day).

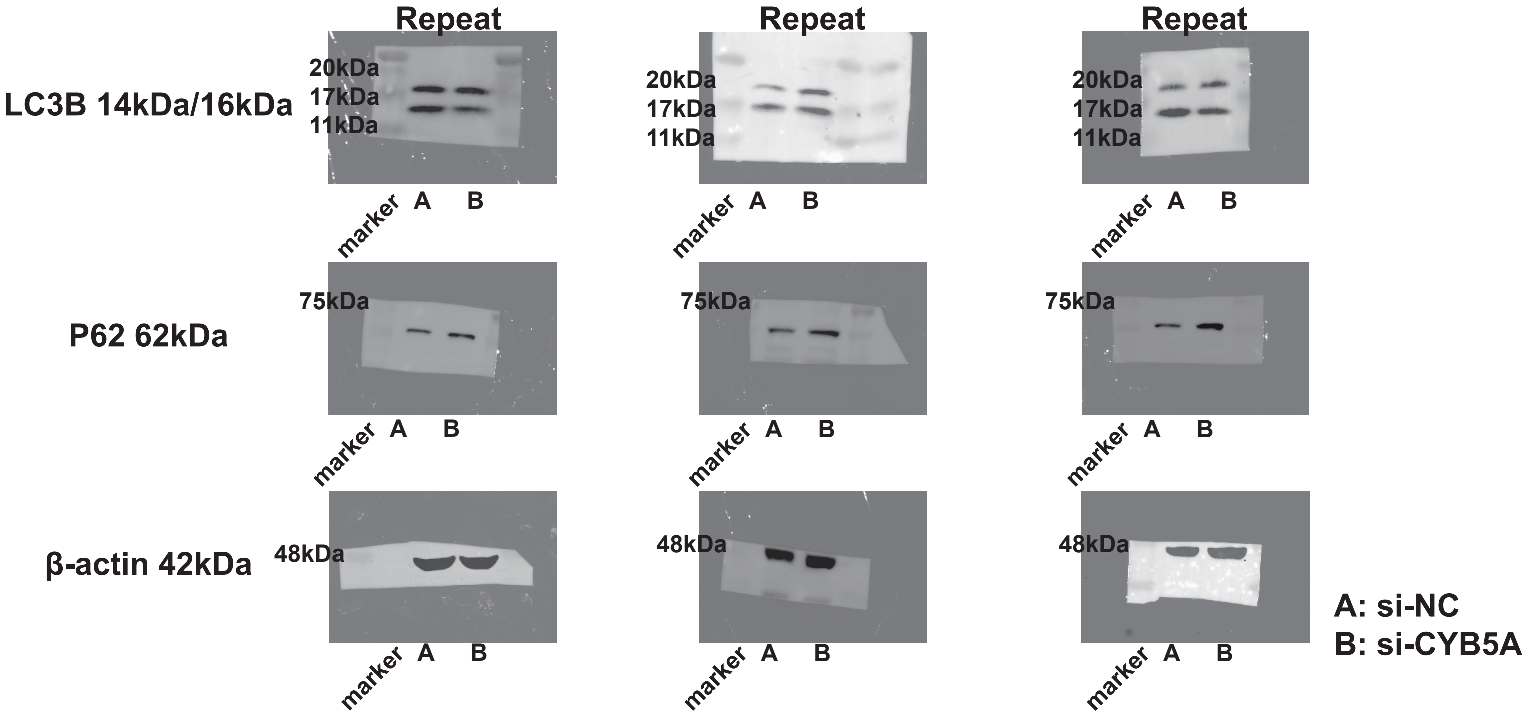

Supplementary Figure 17. Unprocessed Western blotting. Source Data for Figure 4B (14day).

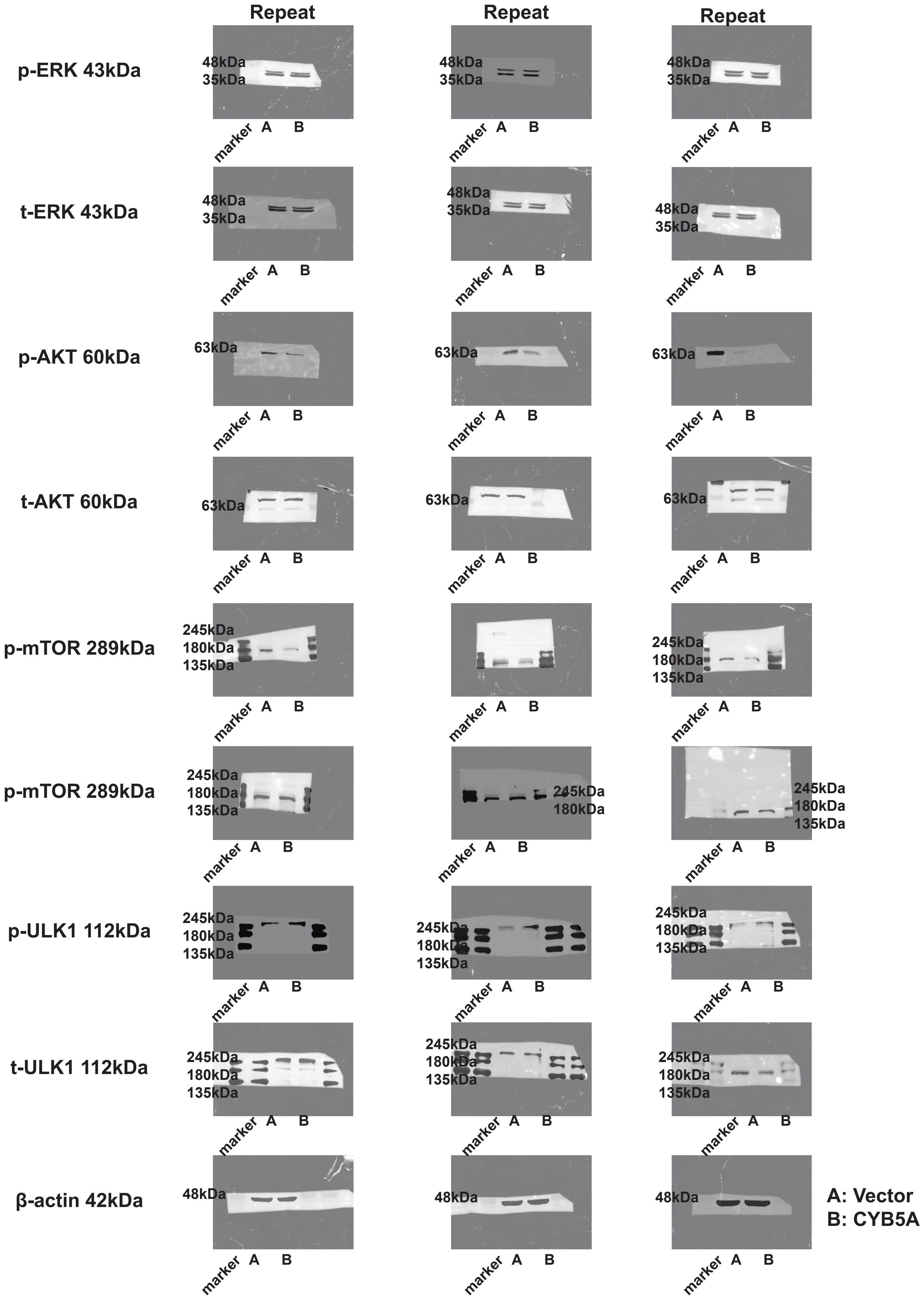

Supplementary Figure 18. Unprocessed Western blotting. Source Data for Figure 4C.

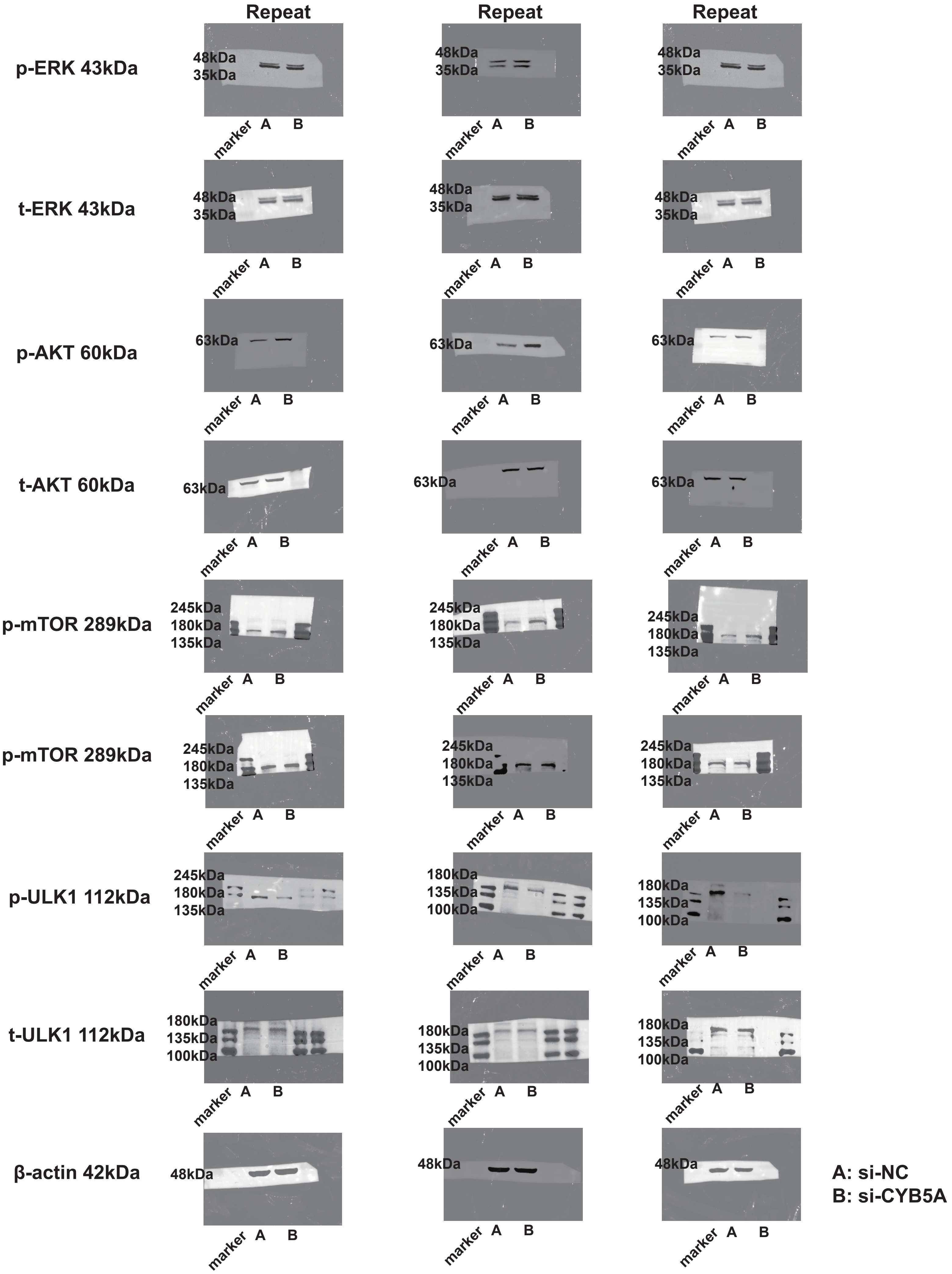

Supplementary Figure 19. Unprocessed Western blotting. Source Data for Figure 4D.

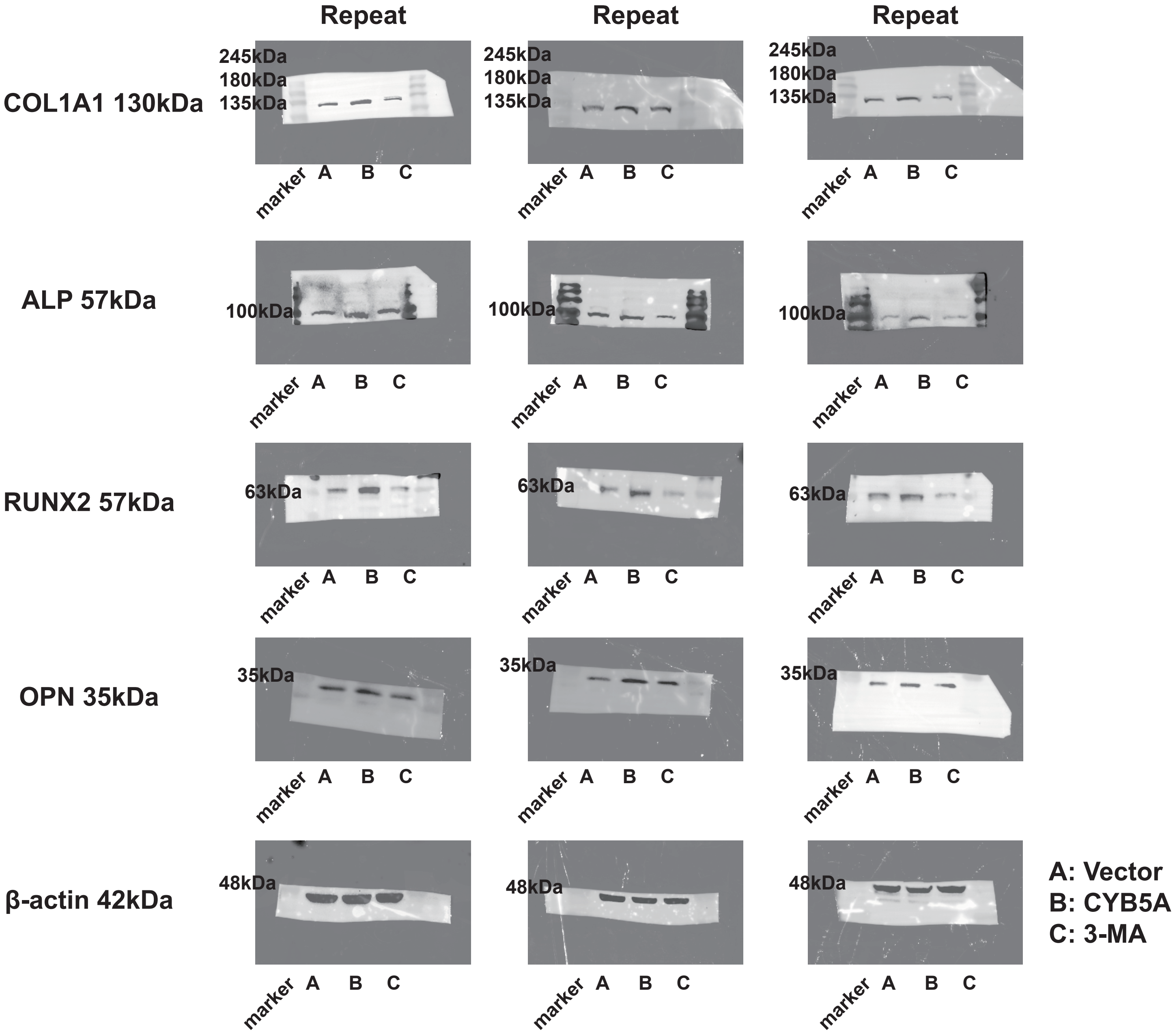

Supplementary Figure 20. Unprocessed Western blotting. Source Data for Figure 5A.

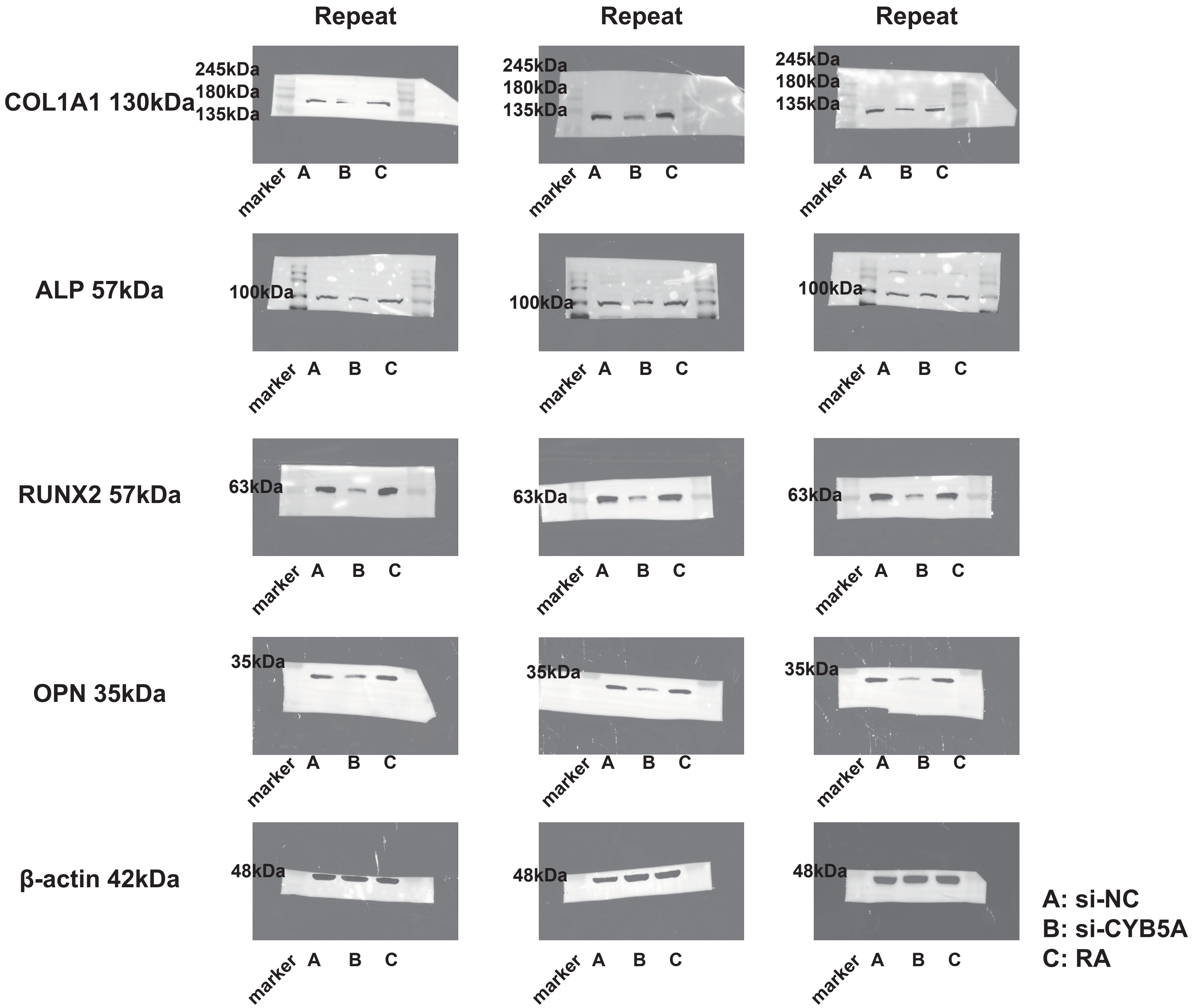

Supplementary Figure 21. Unprocessed Western blotting. Source Data for Figure 5D.

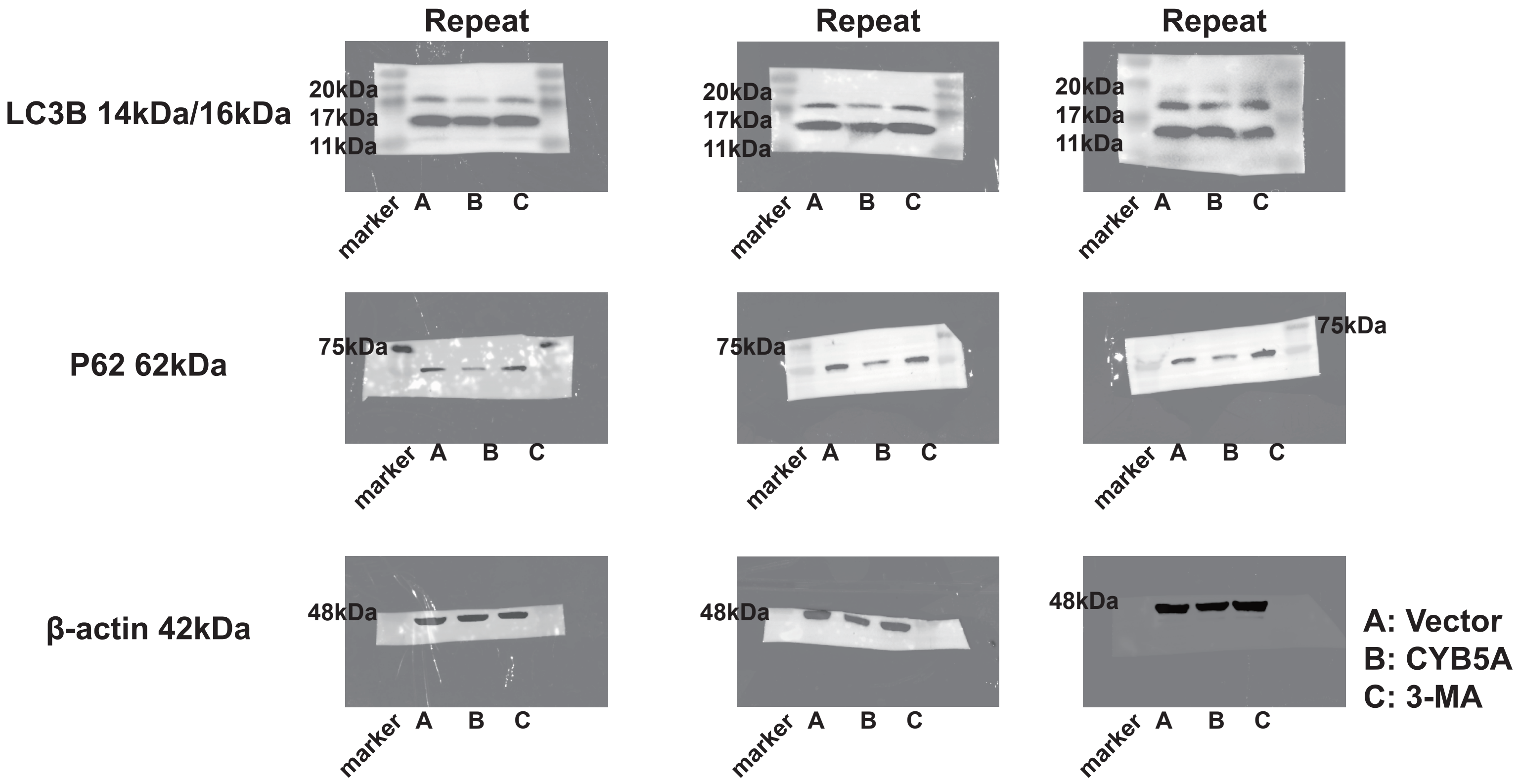

Supplementary Figure 22. Unprocessed Western blotting. Source Data for Figure 5G.

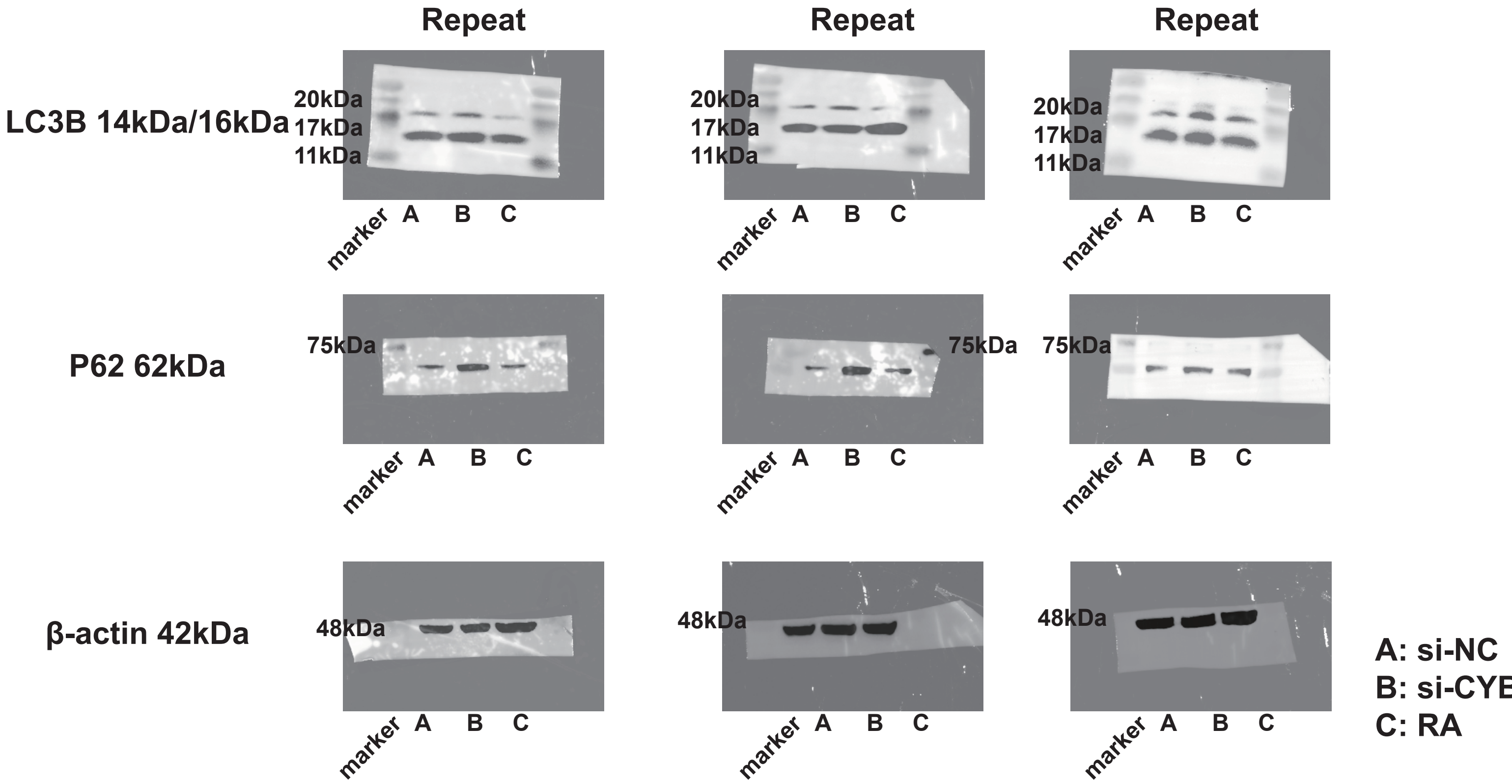

Supplementary Figure 23. Unprocessed Western blotting. Source Data for Figure 5H.

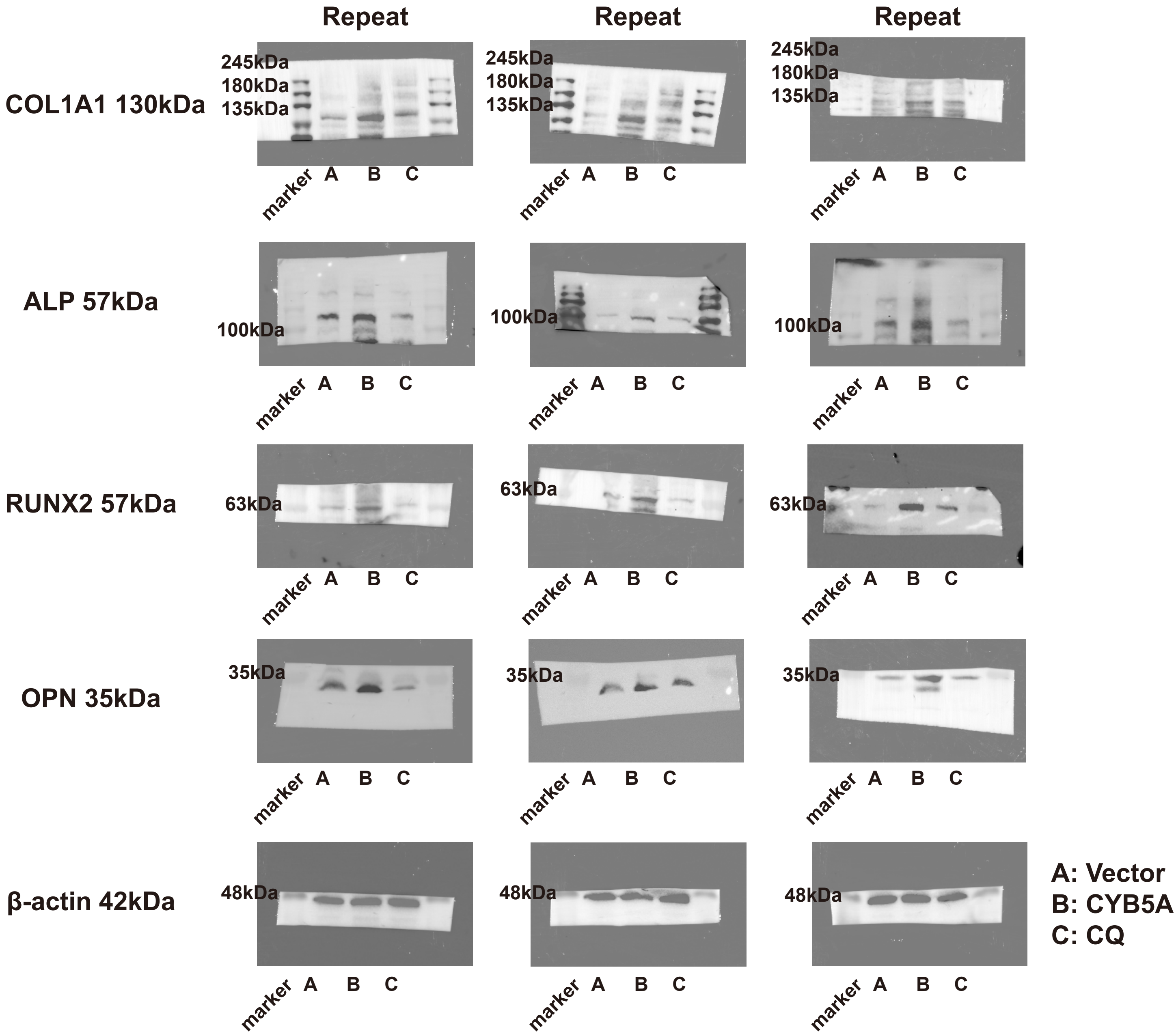

Supplementary Figure 24. Unprocessed Western blotting. Source Data for Figure 5I.

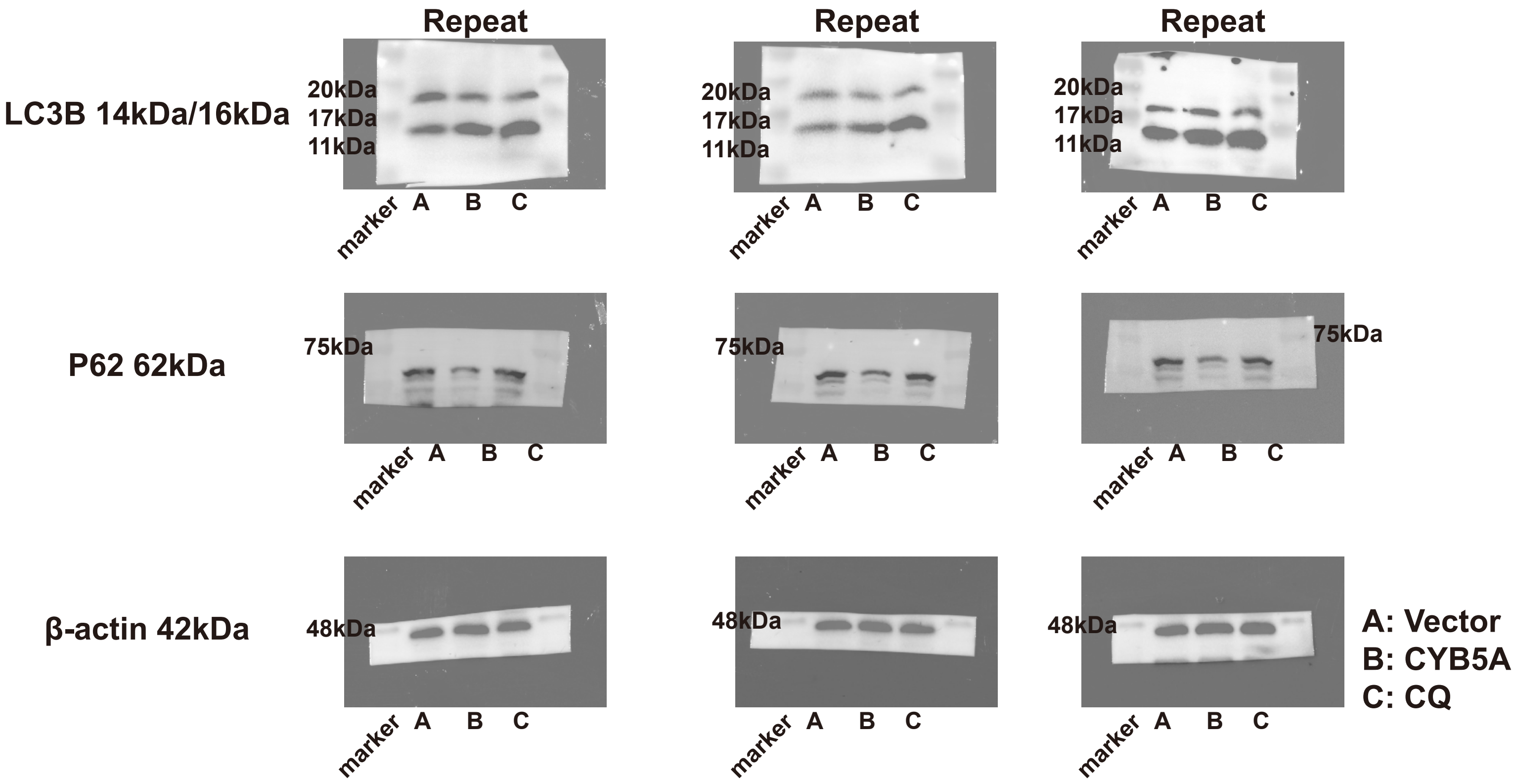

Supplementary Figure 25. Unprocessed Western blotting. Source Data for Figure 5J.

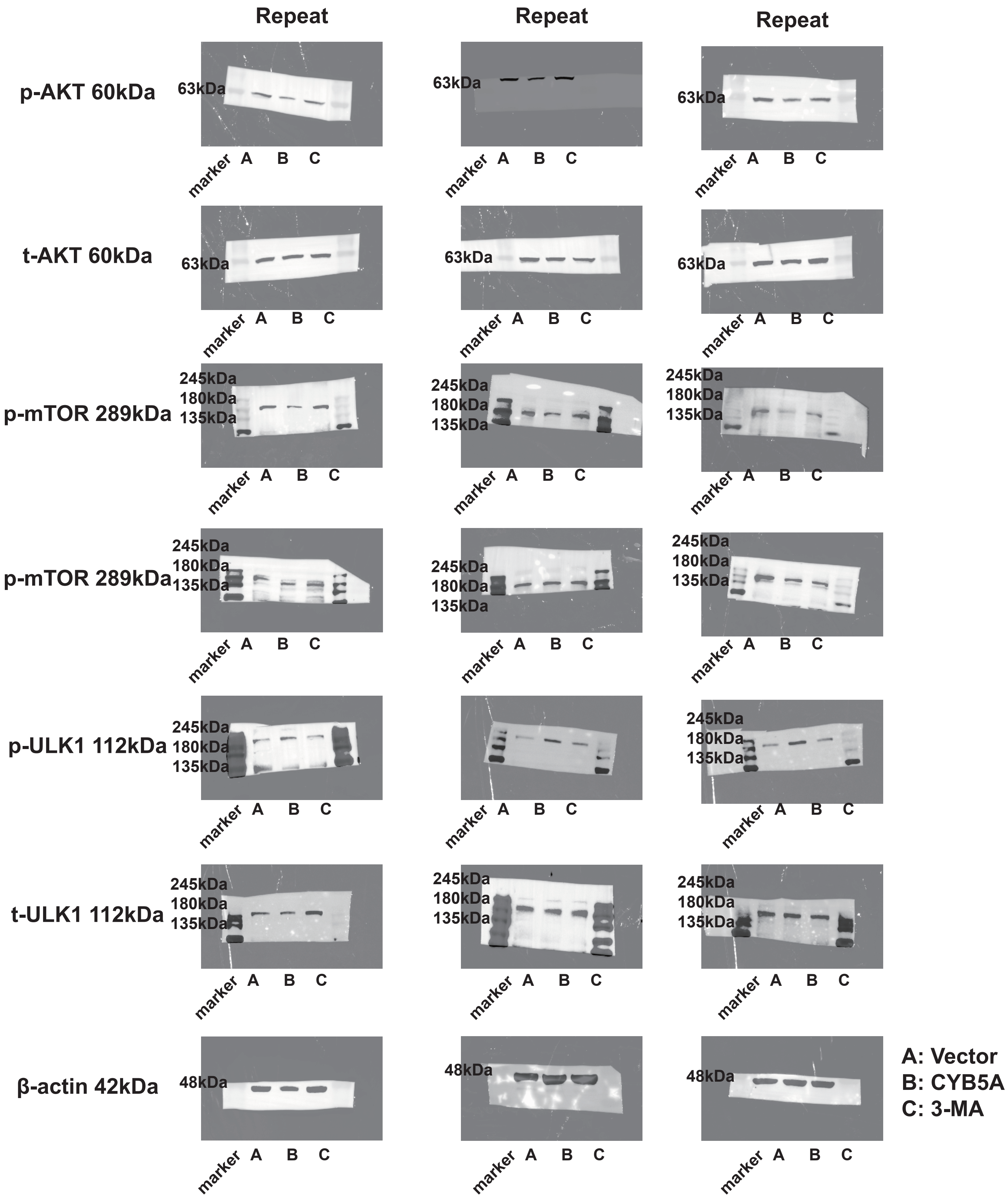

Supplementary Figure 26. Unprocessed Western blotting. Source Data for Figure 6A.

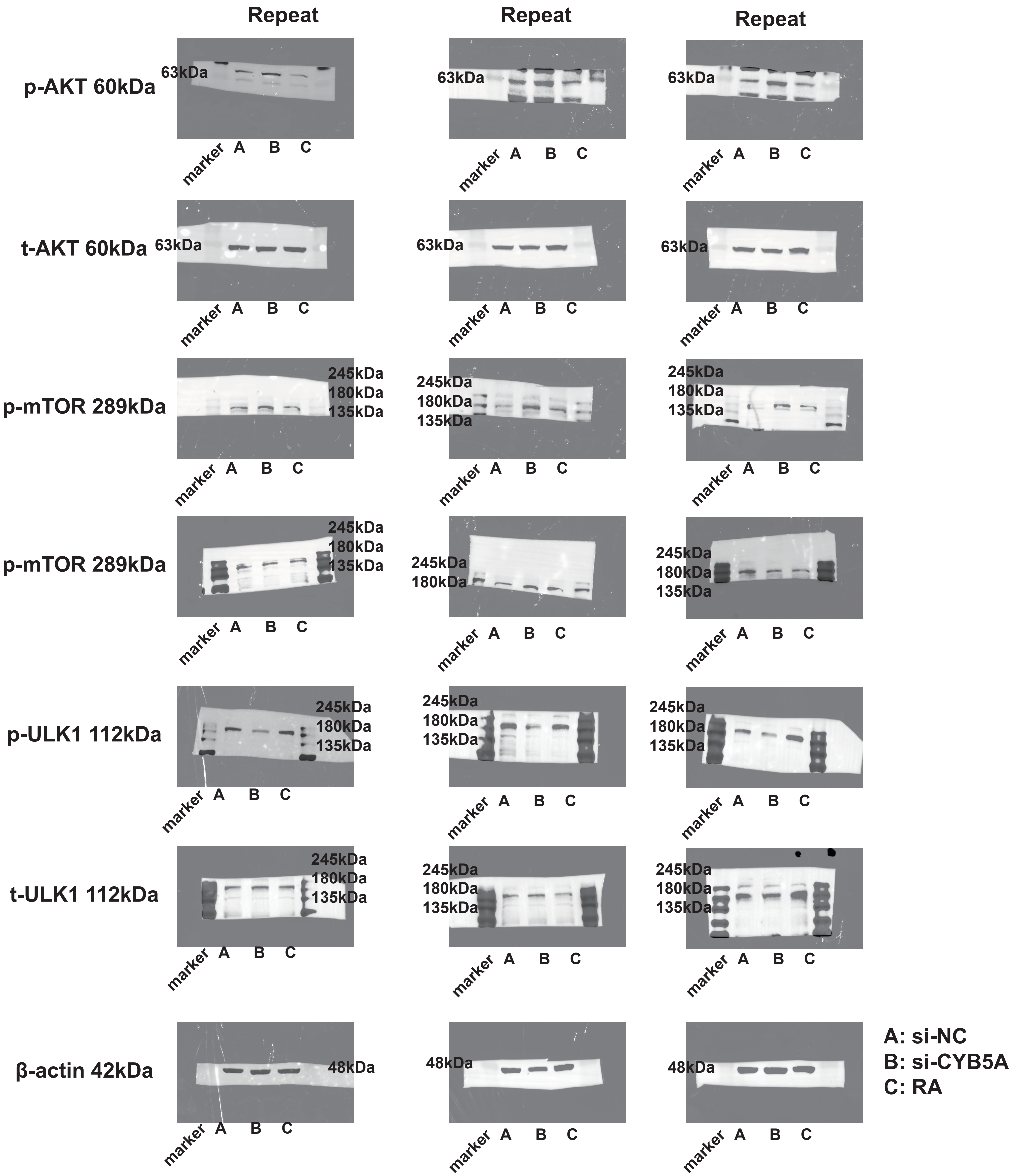

Supplementary Figure 27. Unprocessed Western blotting. Source Data for Figure 6B.
